# Improved locomotor recovery after contusive spinal cord injury in *Bmal1*^−/−^ mice is associated with protection of the blood spinal cord barrier

**DOI:** 10.1038/s41598-020-71131-6

**Published:** 2020-08-26

**Authors:** Lukasz P. Slomnicki, Scott A. Myers, Sujata Saraswat Ohri, Molly V. Parsh, Kariena R. Andres, Julia H. Chariker, Eric C. Rouchka, Scott R. Whittemore, Michal Hetman

**Affiliations:** 1 Kentucky Spinal Cord Injury Research Center, University of Louisville, Louisville, KY 40202 USA; 2 Department of Neurological Surgery, University of Louisville, Louisville, KY 40202 USA; 3 Department of Pharmacology and Toxicology, University of Louisville, Louisville, KY 40202 USA; 4 Department of Anatomical Sciences and Neurobiology, University of Louisville, Louisville, KY 40202 USA; 5 Department of Computer Engineering and Computer Science, University of Louisville, Louisville, KY 40202 USA; 6 Kentucky Biomedical Research Infrastructure Network Bioinformatics Core, University of Louisville, Louisville, KY 40202 USA; 7 Kentucky Spinal Cord Injury Research Center, University of Louisville, 511 S. Floyd St., MDR616, Louisville, KY 40292 USA

**Keywords:** Blood-brain barrier, Diseases of the nervous system, Spinal cord injury

## Abstract

The transcription factor BMAL1/ARNTL is a non-redundant component of the clock pathway that regulates circadian oscillations of gene expression. Loss of BMAL1 perturbs organismal homeostasis and usually exacerbates pathological responses to many types of insults by enhancing oxidative stress and inflammation. Surprisingly, we observed improved locomotor recovery and spinal cord white matter sparing in *Bmal1*^*−/−*^ mice after T9 contusive spinal cord injury (SCI). While acute loss of neurons and oligodendrocytes was unaffected, *Bmal1* deficiency reduced the chronic loss of oligodendrocytes at the injury epicenter 6 weeks post SCI. At 3 days post-injury (dpi), decreased expression of genes associated with cell proliferation, neuroinflammation and disruption of the blood spinal cord barrier (BSCB) was also observed. Moreover, intraspinal extravasation of fibrinogen and immunoglobulins was decreased acutely at dpi 1 and subacutely at dpi 7. Subacute decrease of hemoglobin deposition was also observed. Finally, subacutely reduced levels of the leukocyte marker CD45 and even greater reduction of the pro-inflammatory macrophage receptor CD36 suggest not only lower numbers of those cells but also their reduced inflammatory potential. These data indicate that *Bmal1* deficiency improves SCI outcome, in part by reducing BSCB disruption and hemorrhage decreasing cytotoxic neuroinflammation and attenuating the chronic loss of oligodendrocytes.

## Introduction

Contusive spinal cord injury (SCI), which represents most clinical SCI cases, involves the primary injury and a secondary injury cascade that progresses hours to months post-SCI^1^. Secondary injury is mediated by multiple pathophysiological mechanisms, exacerbating the injury and leading to greater functional loss^1^. In thoracic contusive SCI, functional deficits are driven by white matter (WM) damage^2^. Acute loss of axons and oligodendrocytes (OLs), neuroinflammation, protracted loss of OLs and insufficient myelin repair are major, functionally meaningful components of the SCI-associated WM damage^3^. Oxidative stress is a major contributor to WM injury by driving neurotoxic neuroinflammation and directly causing OL death^4,5^. SCI-activated inflammatory cells including microglia/macrophages are a major source of reactive oxygen species (ROS) and pro-oxidant cytokines that kill OLs^3,4,6^. At least in rodents, lost OLs are robustly replaced by OL precursor cell (OPC)-mediated oligodendrogenesis^3^. However, in spared white matter of the injury epicenter region, lower OL numbers persist chronically while new myelin does not support full recovery of axonal function^7,8^. SCI-associated hemorrhage and disruption of the blood-spinal cord barrier (BSCB) provide major stimuli that: (i) initiate/potentiate neuroinflammation and oxidative stress, (ii) promote axonal regeneration-inhibitory scarring, (iii) impair axonal repair and OL differentiation, and (iv) increase OPC proliferation^3,9,10^.

Transcription factors (TFs) including NFκB and NRF2/NFE2L2 are key regulators of inflammation and oxidative stress response^11,12^. After SCI, NFκB drives expression of pro-inflammatory genes and its inhibition improves outcome of contusive SCI^13^. NRF2 mediates expression of anti-oxidant enzymes in response to oxidative stress and opposes the pro-inflammatory activity of NFκB^11,12^. After contusive SCI, WM sparing (WMS) and functional recovery are enhanced by pharmacological activation of NRF2 while its genetic deletion has opposite effects^14,15^.

Aryl hydrocarbon receptor nuclear translocator like (ARNTL)/brain and muscle ARNT-like 1 (BMAL1) is a basic helix loop helix TF that together with its partners CLOCK or NPAS2 mediates circadian oscillations of gene expression^16^. The genes for core components of the circadian oscillator pathway (the clock pathway) including the TFs *Per1/2*, *Cry1/2, Dbp1* and *Nr1d1/2* are targets of BMAL1:CLOCK/NPAS2 heterodimers. Other target genes are tissue-specific and underlie circadian oscillation of metabolism, immunity/inflammation and anti-oxidant defenses^11,17–20^.

The clock pathway is active in most cells throughout the body and undergoes circadian entrainment by external time cues such as light or feeding^16^. While the suprachiasmatic nucleus (SCN) imposes central circadian rhythmicity throughout the body, local activity of the clock pathway determines homeostasis and injury responses by regulating genes in a tissue-specific manner^21^. BMAL1 is the only non-redundant component of the positive arm of the clock pathway^16^ and its deletion affects inflammation^11,22–26^, anti-oxidant defenses^11,20^, hemostasis^27–29^ and vascular function^30^. Consequently, loss of BMAL1 is usually pro-pathogenic. Germ line *Bmal1* knock out (KO) mice show accelerated aging^31^. Germ line-, or various cell type-specific, *Bmal1* KOs worsened outcome in several disease models including high fat diet-induced obesity and atherosclerosis^22,25,32^, cardiac hypertrophy^33^, sepsis^22–24^, alcohol-induced liver disease^34^, multiple sclerosis-like experimental autoimmune encephalomyelitis (EAE)^26^, and Alzheimer’s disease-like β-amyloidosis^35^. Enhanced acute toxic injury of endothelial cells (ECs) or neurons was also reported^20,36^.

Pro-homeostatic and injury-adaptive effects of BMAL1 are mediated by its regulatory contributions to TF networks that co-ordinate metabolism, innate or adaptive immunity as well as response to stress. BMAL1 opposes NFκB-mediated positive regulation of various pro-inflammatory mediators including the SCI-induced chemokine CCL2^19,22^. In addition, it directly stimulates expression of NRF2, thereby attenuating NFκB- and HIF1α-mediated, pro-neuroinflammatory gene expression in macrophages^11^. In neurons, BMAL1 co-activates NRF2 target genes, increasing resistance to oxidative stress^20^. Therefore, loss of *Bmal1* could worsen the outcome of contusive SCI by enhancing oxidative stress and neuroinflammation. The current study was designed to test such a possibility by determining the extent and potential mechanism(s) underlying functional recovery after T9 moderate contusive SCI in *Bmal1*^*−/−*^ mice.

## Results

### The clock pathway is dysregulated acutely after SCI

**Figure 1:**
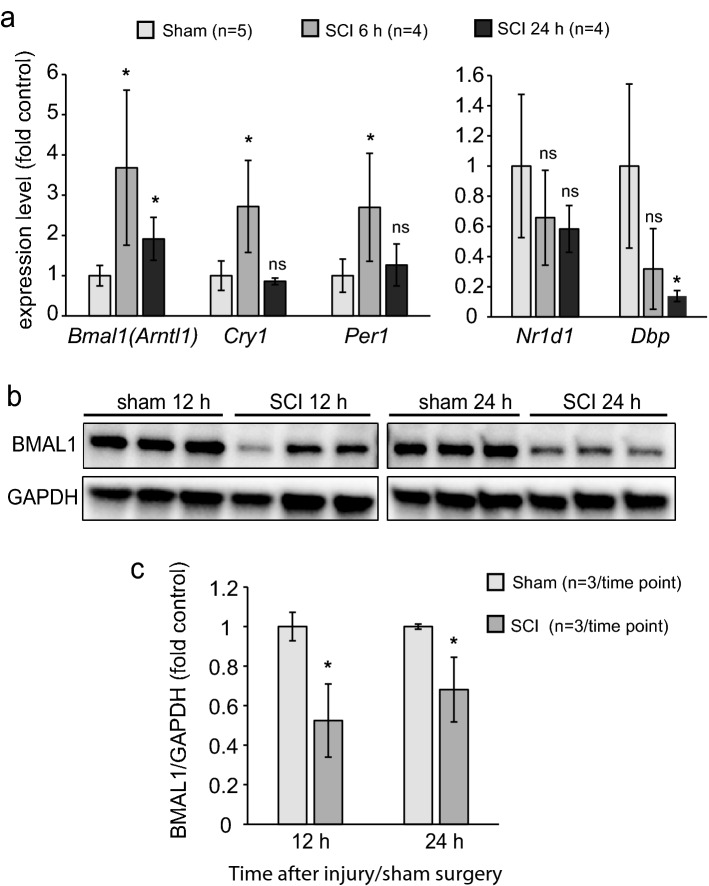
Acute dysregulation of the clock pathway in the contused spinal cord. WT female mice were subject to SCI (T9 IH contusion, 50 kdyn) or sham surgery and sacrificed at the indicated times. Total RNA (**a**) or proteins (**b**, **c**) were extracted from a 5 mm spinal cord segment that included the injury epicenter and the penumbra. (**a**) Expression of the clock pathway genes was analyzed by qPCR. Sham controls sacrificed at 6 (n = 2) or 24 h (n = 3) were pooled together as they showed little variability for the tested Clock pathway transcripts. After SCI, *Bmal1* and its target genes *Cry1* and *Per1* were upregulated. However, two other BMAL1 target genes *Dbp1* and *Nr1d1* showed an opposite trend or no change, respectively. (**b**, **c**) Western blot analysis revealed reduced levels of BMAL1 protein. Untrimmed western blot images are shown in Supplementary Fig. S10. Data are mean ± SD (*p < 0.05; ns, p > 0.05, *u*-test).

TFs that form the clock pathway are by themselves regulated at the transcriptional level^16^. Therefore, mRNA levels for several components of the clock pathway were determined in a 5 mm segment of the contused spinal cord tissue spanning the injury epicenter and penumbra. Expression of *Bmal1* was up at 6 and 24 h after SCI (Fig. 1a). Other clock pathway genes that are directly regulated by BMAL1 were also affected. Mediators of the fast feedback inhibition of BMAL1 *Cry1* and *Per1* were up at 6, but not 24 h, post-injury (Fig. 1a). Conversely, the BMAL1 target gene *Dbp1* displayed the opposite trend which reached significance 24 h after SCI while *Nr1d1* mRNA levels were unchanged (Fig. 1a). Those findings suggest SCI associated dysregulation of the clock pathway.

**Figure 2:**
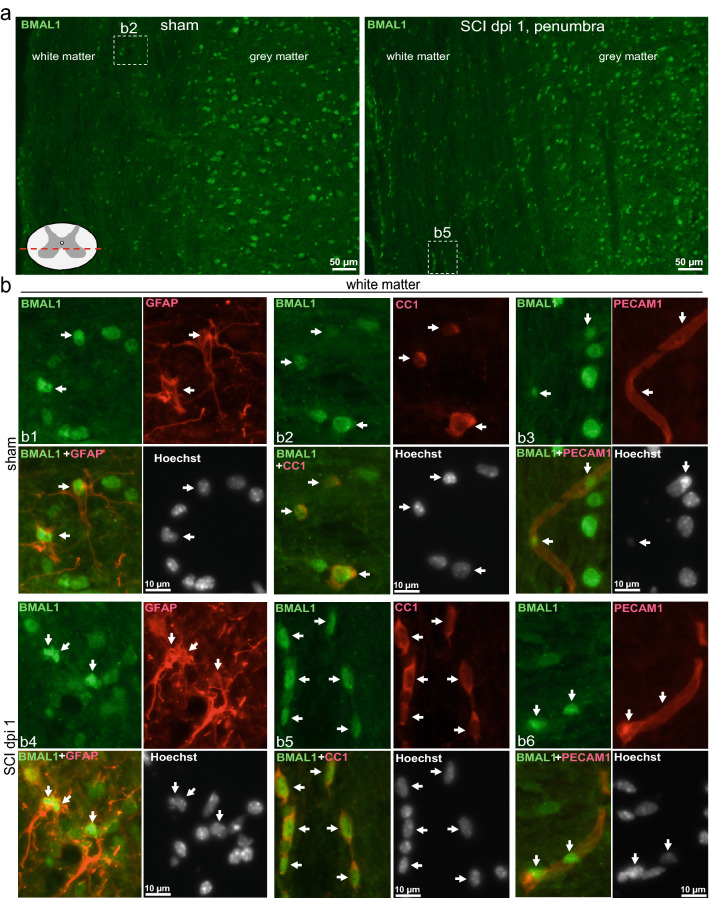
Expression of BMAL1 in white matter cells. Sham or SCI mice were killed at ZT 3–5. Co-immunostaining for BMAL1 and the indicated cell type-specific markers were performed on longitudinal (coronal) sections that included the injury epicenter and penumbral regions; nuclei were counterstained with Hoechst-33258. The specificity of the BMAL1 signal was validated by showing its loss in *Bmal1*^*−/−*^ spinal cord tissue or after replacing the anti-BMAL1 antibody with a control IgG (Supplementary Fig. S1). As acute cell loss in the injury epicenter may affect identification of BMAL1-expressing cells, all images from SCI animals depict the penumbral region (0.5–1 mm rostral from the injury epicenter). (**a**) Nuclear BMAL1 signal is present in numerous cells throughout the grey and white matter with similar patterns in sham and SCI mice. The sectioning level is indicated by the red line. The images depict the ventral horn grey matter and the ventrolateral white matter after co-staining for BMAL1 and the OL marker CC1. Position of high power images that are shown in (**b**) is indicated. (**b**) High power images of BMAL^+^ white matter cells that were co-stained for the OL marker CC1, the astrocyte marker GFAP and the EC marker PECAM1. Arrows point double positive cells. All co-immunostainings were done on adjacent sections that were cut as in (**a**); low power images are shown in Supplementary Figs. S2–S5. The images represent analysis of 3 sham and 3 SCI WT female mice.

**Figure 3:**
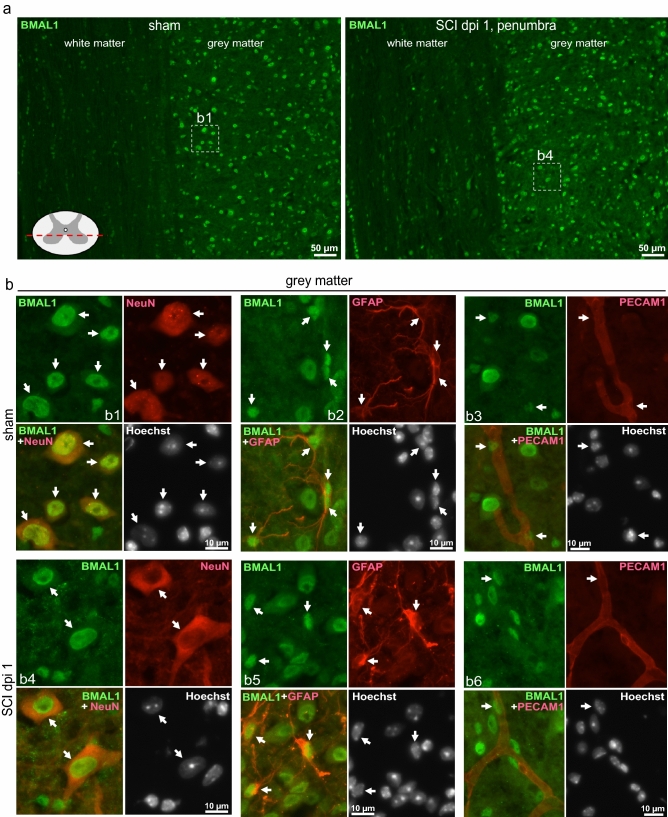
Expression of BMAL1 in grey matter cells. Animals, co-immunostaining and imaging were as described for Fig. 2. (**a**) Representative images of BMAL1 signal in the ventral horn grey matter and the ventrolateral white matter after co-staining for the neuronal marker NeuN. The sectioning level is indicated by the red line. The position of higher power images that are shown in (**b**) is indicated. (**b**) High power images of BMAL^+^ grey matter cells that were co-stained for the neuronal marker NeuN, the astrocyte marker GFAP and the EC marker PECAM1. Arrows point double positive cells. All co-immunostainings were done on adjacent sections that were cut as in (**a**); low power images are shown in Supplementary Figs. S2–S5.

This dysregulation was accompanied by reduced expression of BMAL1 protein that at 24 h after injury showed 65% of sham control values as determined by western blot analysis (Fig. 1b,c). Acute loss of neurons in the injury epicenter is a likely contributor to such a decline as immunofluorescence analysis showed strong neuronal BMAL1 signal in sham control spinal cord tissue or the injury penumbra region (Figs. 2, 3). Therefore, to avoid confounding effects of acute cell loss, identification of spinal cord cells that express BMAL1 was performed in the penumbra 0.5–1.0 mm rostral from the injury epicenter. At 24 h after sham surgery or SCI, nuclear BMAL1 immunofluorescence was observed in cells throughout the grey matter (GM) and the WM with GM neurons showing the strongest signal (Figs. 2, 3, Supplementary Figs. S1–S5). In the WM, BMAL1 was expressed in CC1^+^ OLs and GFAP^+^ astrocytes (Fig. 2, Supplementary Figs. S3, S4). In the GM, NeuN^+^ neurons showed a strong BMAL1 staining and BMAL1^+^ astrocytes were also ubiquitous (Fig. 2, Supplementary Figs. S2, S3). In sham samples, GM OLs were BMAL1^+^, but their identification after SCI was not possible due to the appearance of a positive CC1 signal in neurons (Supplementary Fig. S4). Occasional PECAM^+^/BMAL1^+^ endothelial cells (ECs) were also observed in WM and GM (Figs. 2, 3, Supplementary Fig. S5). IBA1^+^ microglia/macrophages did not express detectable BMAL1. Hence, SCI-associated dysregulation of the clock pathway may occur in various types of spinal cord cells including neurons, astrocytes, OLs, and ECs.

### Improved locomotor function after SCI in *Bmal1*^*-/-*^ mice

**Figure 4:**
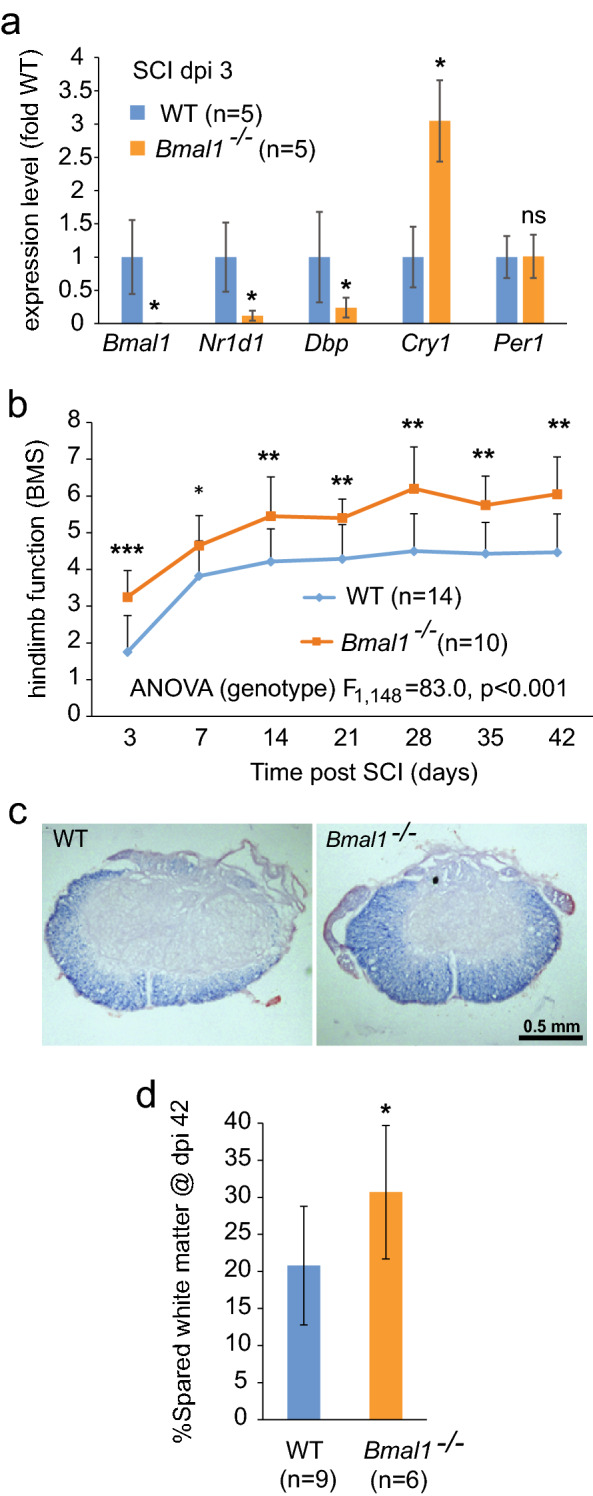
Improved locomotor recovery and increased white matter sparing (WMS) in *Bmal1*^*−/−*^ mice with SCI. (**a**) WT and *Bmal1*^*−/−*^ mice (M:F = 1:4 in each group) underwent SCI and were killed on dpi 3. Clock pathway transcripts were analyzed in the injury epicenter/penumbra region by qPCR. In *Bmal1*^*−/−*^ mice, *Bmal1* mRNA was undetectable while *Nr1d1* and *Dbp1* mRNAs were reduced and *Cry1* upregulated. (**b**) WT (M:F = 7:7, including 3 KO littermates) and *Bmal1*^*−/−*^ (M:F = 3:7) mice underwent SCI. BMS analysis of hindlimb function revealed improved chronic recovery in the *Bmal1*^*−/−*^ group. (**c**, **d**) WMS analysis was performed on spinal cord tissue collected after completion of the last BMS assessment on dpi 42 (WT M:F = 5:4, *Bmal1*^*−/−*^ M:F = 2:4). (**c**) Representative images of eriochrome cyanine (EC)-stained transverse sections through the injury epicenter. Note the destruction of EC-stained myelin in the dorsal column area and its relative sparing in the ventral white matter. (**d**) In the injury epicenter, quantification of EC^+^ myelin signal revealed increased white matter sparing in *Bmal1*^*−/−*^ mice. Data are the mean ± SD; *p < 0.05, **p < 0.01, ***p < 0.001—*u*-test in (a, d), RM ANOVA with Tukey post*-hoc* tests in (**b**) (factor 1, genotype: F_1,148_ = 83.0, p < 0.001; factor 2, time of recovery: F_6,38_ = 27.5, p < 0.001; factor 1 × factor 2: F_6,38_ = 0.59, p > 0.05). No significant gender effects were observed when sex was included as an additional factor in BMS data analysis (gender: F_1,133_ = 0.02, p > 0.05, gender x genotype: F_1,133_ = 0.77, p > 0.05, gender x genotype x time of recovery: F_6,35_ = 0.64, p > 0.05).

To evaluate the role of BMAL1 in SCI pathogenesis, *Bmal1*^*−/−*^ mice were used. Relative to WT at 3 days post injury (dpi), contused *Bmal1*^*−/−*^ spinal cord tissue had undetectable *Bmal1*, and reduced *Nr1d1* and *Dbp1* mRNAs, confirming disruption of the clock pathway (Fig. 4a). Malfunction of the clock pathway fast feedback inhibition loop is suggested by increased *Cry1* mRNA (Fig. 4a). Surprisingly, *Bmal1*^*−/−*^ mice showed markedly improved locomotor recovery (Fig. 4b). Final BMS scores at dpi 42 were 6.05 ± 1.01 and 4.46 ± 1.05 for *Bmal1*^*−/−*^ and WT animals, respectively. That score difference represents major locomotor improvements including the presence of weight bearing, coordinated, plantar stepping in *Bmal1*^*−/−*^ mice^37^. Importantly, increased WMS was observed at the injury epicenter of *Bmal1*^*−/−*^ mice (Fig. 4c, d). Therefore, enhanced post-SCI recovery of hindlimb function in *Bmal1*^*−/−*^ mice is likely mediated by WM protection as shown previously in contusive T9 SCI^2^.

### Attenuated loss of OLs in *Bmal1*^*−/−*^ mice

**Figure 5:**
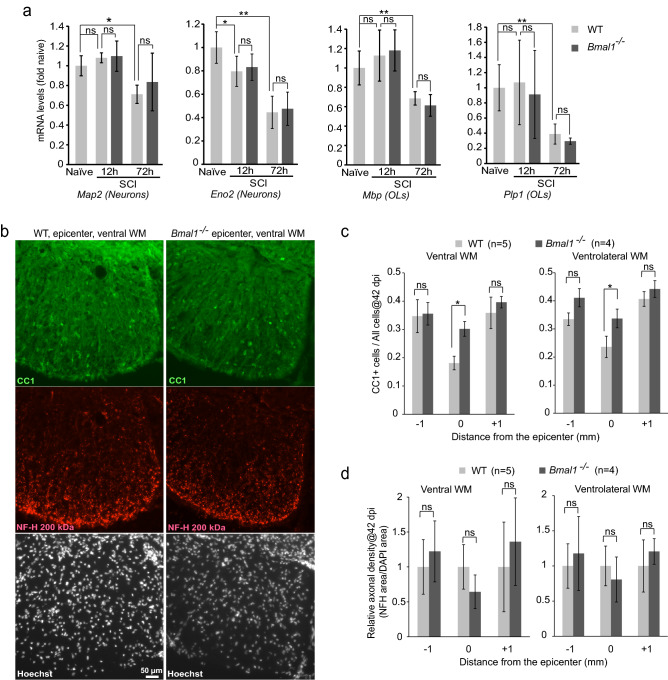
Decreased SCI-associated OL loss in *Bmal1*^*−/−*^ mice. (**a**) SCI WT and *Bmal1*^*−/−*^ mice were as described for Fig. 4a (n = 5/genotype). Naïve WT controls received the same treatment as SCI mice except surgery and were killed after 3 days (n = 5, M:F = 2:3). Expression of mRNAs specific for OLs or neurons was analyzed by qPCR. Both OL and neuronal markers declined at dpi 3, indicating acute loss of those cells. Similar declines in WT and *Bmal1*^*−/−*^ mice suggest no effects of the *Bmal1*^*−/−*^ genotype on acute loss of OLs or neurons. (**b–d**) SCI WT and *Bmal1*^*−/−*^ mice were as described for Fig. 4b (WT M:F = 2:3 including 3 KO littermates, *Bmal1*^*−/−*^ M:F = 1:3). At dpi 42, after completion of the last BMS evaluation, mice were sacrificed. Transverse sections were co-immunostained to detect OLs (CC1) and axons (anti-NFH); cell nuclei were counterstained with Hoechst-33258. (**b**) Representative images of that staining in the ventral WM. (**c**) In the ventral and ventrolateral WM at the injury epicenter, the decline of OL density (CC1^+^/Hoechst^+^ cells) was attenuated in *Bmal1*^*−/−*^ mice suggesting reduced chronic loss of OLs (RM ANOVA, factor: genotype, F_1,7_ = 10.245 or 26.026, p < 0.05 for ventral or ventrolateral WM, respectively). (**d**) Relative axonal density (defined as NFH staining area to Hoechst staining area normalized to WT) was unaffected. (RM ANOVA, factor genotype, F_1, 7_ = 0.0786 or 0.123, p > 0.05 for ventral or ventrolateral WM, respectively). Data in (**a**, **c**, **d**) are means ± SD (*p < 0.05; **p < 0.01, ns, p > 0.05, *u*-test in (**a**), Tukey post*-hoc* tests in (**c**, **d**).

Increased WMS may be a result of reduced loss of OLs and/or attenuated loss of axons. After mouse contusive SCI, most OLs are lost in the injury epicenter at 24 h post-SCI^38^. However, *Bmal1*^*−/−*^ and WT mice showed similar declines in epicenter OL-specific mRNAs at dpi 3 (Fig. 5a). Similar declines of neuronal mRNAs suggested no effects of *Bmal1* deletion on acute loss of neurons either (Fig. 5a).

As OL loss continues beyond dpi 3, leading to maximum decline of OL number in the epicenter region at 4 weeks after injury^7,38,39^, the relative content of OLs was quantified in the injured spinal cord of *Bmal1*^*−/−*^ and WT mice at dpi 42. This analysis focused on the ventral and ventrolateral WM that in rodents are the principal anatomical substrates of hindlimb function recovery^40,41^. The OL fraction, defined as % CC1^+^/Hoechst 33258-stained cells, declined in the injury epicenter as compared to sections 1 mm rostral or caudal from that region (Fig. 5b,c). Consistent with a previous report^7^, WT mice showed a 40–50% decline of relative OL content (Fig. 5b,c). Those decreases were reduced to 20–25% in *Bmal1*^*−/−*^ mice (Fig. 5b,c). Therefore, BMAL1 deficiency attenuated the chronic SCI-induced loss of OLs. Such an effect could reflect decrease in protracted OL death and/or increased OL replacement that both influence WM OL content after SCI^3^.

Conversely, in the ventral/ventrolateral WM, relative axonal density was unaffected (Fig. 5b,d). These findings indicate that greater locomotor recovery in *Bmal1*^*−/−*^ mice is likely due to reduced OL loss and the associated improvement in axonal function rather than primary protection of axons. As small increases in axonal sparing may have remarkable effects on locomotion^3^, it is still possible that functionally meaningful axonal sparing occurred in KOs but was not detected.

### SCI transcriptome changes in *Bmal1*^*−/−*^ mice after SCI suggest attenuated BSCB disruption and reduced neuroinflammation

**Figure 6:**
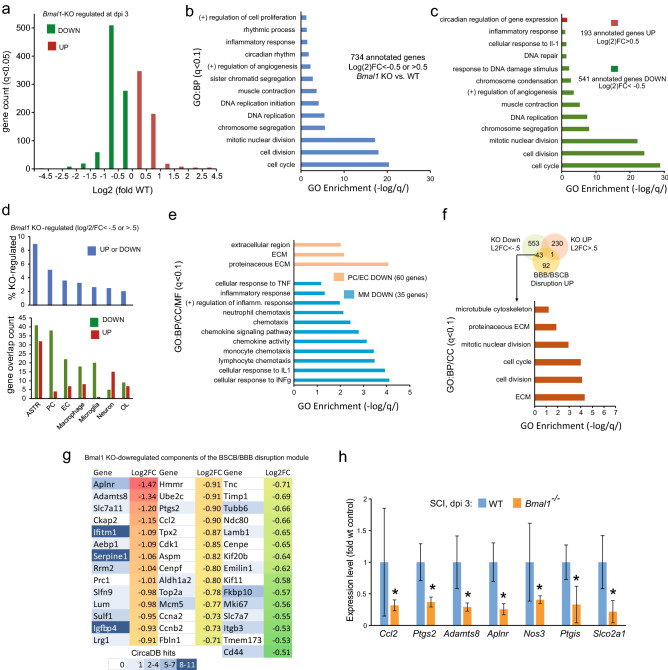
*Bmal1*^*-/-*^ mediated changes in the SCI transcriptome suggest attenuated disruption of the BSCB and reduced neuroinflammation. RNA from dpi 3 contused spinal cord tissue of WT (M:F = 2:2, including 1 KO littermate) and *Bmal1*^*−/−*^ (M:F = 1:2) mice was analyzed by RNASeq (a-g) or qPCR (h). (**a**) Gene count of *Bmal1*^*−/−*^ effects on the transcriptome (see Supplementary Table S1 for details). (**b**, **c**) Gene ontology (GO) analysis of the highly affected genes in *Bmal1*^*−/−*^ spinal cords. Analysis was run for all genes *(b)* or separately for upregulated and downregulated genes *(c)*. Note the significant enrichment of GO:Biological Process (BP) terms associated with cell proliferation, inflammation and angiogenesis in both analyses. (**d**) Cell type-enriched transcripts in *Bmal1*^*−/−*^ regulated SCI transcriptome. Note the relatively higher representation of astrocyte (ASTR) and vascular cell transcripts (EC + PC) and the predominantly gene downregulation trend for PC-, EC- and macrophage/microglia (MM)-enriched transcripts. Cell type-enriched gene lists are defined in the “Materials and methods”. (**e**) Significant enrichment of GO terms in downregulated MM- or PC/EC-enriched genes. Note the presence of neuroinflammation or ECM/cell proliferation-associated terms in MM or PC/EC genes, respectively. Cellular component (CC) and molecular function (MF) GOs are also included. No significant GO enrichment was observed for KO-regulated astrocyte genes. (**f**) Brain EC genes that are upregulated during BBB/BSCB disruption^44^ are highly represented among *Bmal1*^*−/−*^ downregulated genes. Overlapping genes overrepresent GOs that are associated with BBB/BSCB disruption responses such as synthesis of ECM components and vascular cell proliferation. (**g**) Heat map of *Bmal1*^*−/−*^ downregulated components of the BBB/BSCB disruption module. Note that several of those genes appear to be regulated in a circadian fashion, as shown by the number of experiments detecting their significant circadian oscillations in the CircaDB database (# of CircaDB hits). (**h**) qPCR analysis of selected vascular cell genes that are downregulated in *Bmal1*^*−/−*^ spinal cords. Note the downregulation of genes that likely promote BBB/BSCB failure (*Ccl2*, *Ptgs2*, *Adamts8*) and potentially pro-hemostatic downregulation of *Aplnr*, *Nos3*, and *Ptgis. Slco2a1* is an arachidonic acid transporter that supports activities of *Ptgs2* and *Ptgis*. Data are means ± SD (*p < .05, *u*-test).

RNASeq analysis provided further insight into potential BMAL1 contributions to SCI pathogenesis. Compared to WT, there were 874 KO-downregulated and 578 upregulated genes in the lesion site at dpi 3 (q < 0.05, Fig. 6a, Supplementary Table S1). The highly regulated genes (Log2FC < − 0.5 or > 0.5) were enriched for Gene Ontology terms (GOs) associated with cell proliferation, angiogenesis, inflammation and circadian rhythms (Fig. 6b, q < 0.1). The “circadian regulation of gene expression” was the only significantly enriched GO among the upregulated genes (Fig. 6c). GOs linked to cell proliferation, angiogenesis and inflammation were enriched among the downregulated genes (Fig. 6c). Cell proliferation-related GOs showed maximum enrichment (i.e. lowest q values, Fig. 6c). These findings suggest that loss of *Bmal1* reduces post-injury cell proliferation that may be linked to neuroinflammation, angiogenesis, OL replacement, and/or scar formation.

Further deconvolution of potential KO-associated anti-pathogenic mechanisms was achieved by analyzing KO effects on cell type-enriched transcripts from previously published cell specific transcriptomes (Fig. 6d,e). Those included the top 1,000 highest expressed genes in macrophages from contused thoracic mouse spinal cord at dpi 3, top 500 genes enriched specifically in uninjured mouse brain microglia, myelinating as well as newly formed OLs, astrocytes, and neurons, or 467 mouse brain EC-specific transcripts, or 260 mouse brain pericyte (PC)-specific transcripts^42–45^ (see “Materials and methods” for details). Extensive overlaps exceeding 50 genes were observed for astrocytes and vascular cells (EC/PC) encompassing 8.8 or 8.6% KO-regulated genes, respectively. *Bmal1*^*−/−*^ associated downregulation was the dominant trend for EC/PC (PC: 38 down, 4 up; EC: 22 down, 7 up) while similar numbers of astrocyte-specific transcripts were down- or upregulated (41 or 32, respectively, Fig. 6d). The predominant downregulation for the identified vascular cell transcripts is unlikely related to changes in cell number as vast portions of EC/PC transcriptomes (> 90%) were expressed at similar levels in WT and *Bmal1*^*−/−*^ spinal cords. No GO enrichment was found in the *Bmal1*^*-/-*^ affected astrocyte genes. GOs associated with extracellular matrix (ECM) were enriched among the downregulated EC/PC genes (Fig. 6e). Downregulation prevailed for macrophage/microglia genes (35 down, 9 up, Fig. 6d). The macrophage/microglia down gene GOs were associated with inflammation including cytokine responses, chemotaxis, as well as chemokine activity (Fig. 6e).

BSCB disruption is a major contributor to post-SCI cell proliferation and neuroinflammation^3,9,46^. As transcriptomes of BSCB-forming cells such as EC, PC and astrocytes were affected by *Bmal1* deletion^3,9,46^, expression of the recently identified blood brain barrier (BBB)/BSCB disruption transcripts (the BBB/BSCB disruption module) was examined. Those include 136 brain/spinal cord EC expressed genes whose upregulation is a signature of BBB/BSCB disruption in at least 3 out of 4 mouse models of neurological diseases including status epilepticus, TBI, stroke, or EAE^44^. In *Bmal1*^*−/−*^ mice, 43 of them were downregulated, which represents 31.6% of the module (Fig. 6f,g). The previously reported enrichment for GOs-related to ECM and EC cell proliferation^44^ was still observed in the *Bmal1*^*−/−*^ regulated portion of the module (Fig. 6f). Importantly, it also included the presumed effector contributors to BBB/BSCB disruption such as the matrix metalloproteinase *Adamts8*, the inflammatory mediator *Ptgs2*/*Cox2* and the chemokine *Ccl2*^44^*.* Their downregulation was confirmed by qPCR (Fig. 6g,h). Other qPCR validated gene downregulations, which may be relevant for potential pro-BSCB/anti-hemorrhagic effects in *Bmal1*^*−/−*^ mice, include EC-expressed pro-angiogenic/anti-thrombotic genes *Aplnr*, *Nos3*, and *Ptgis*. Consistent with reductions of *Ptgs2* and *Ptgis,* which use arachidonic acid to produce prostaglandins, the arachidonic acid transporter *Slco2a1* was also downregulated. The only *Bmal1*^*−/−*^ upregulated gene of the BBB/BSCB disruption module is the core component of the clock pathway, the TF *Nfil3* (Supplementary Table S1). The presence of *Nfil3* in the BBB/BSCB module suggests direct links between the BSCB and clock-associated transcription. Indeed, using the circaDB database (https://circadb.hogeneschlab.org/about), we found 24 of 43 *Bmal1*^*−/−*^ downregulated components of the BSCB disruption module to undergo circadian mRNA oscillations in at least one mouse tissue (Fig. 6g).

We observed an extensive overlap of *Bmal1*^*−/−*^ regulated genes with the top 500 OPC-specific genes from uninjured mouse brain (64 down, 18 up, Supplementary Fig. S6)^43^. However, such overlap may be falsely biased towards OPCs as proliferation-associated genes are enriched in OPCs in the uninjured CNS (Supplementary Fig. S6). Indeed, OPC down-regulated genes show high enrichment of cell proliferation-associated GOs (e.g. 27 out of 60 OL annotated genes are cell cycle-related, Supplementary Fig. S6). SCI at dpi 3 activates the proliferation of OPCs, microglia/macrophages, ECs, PCs and perivascular fibroblasts^5,38,47–49^. Hence, those “OPC” gene changes may also reflect anti-proliferative effects of *Bmal1* deletion in other cell populations.

Collectively, the transcriptomic analysis revealed that in *Bmal1*^*−/−*^ mice, reduced WM damage and enhanced locomotor recovery may be associated with pro-vascular and anti-inflammatory effects including improved function of the BSCB.

### *Bmal1* KO-associated attenuation of post-SCI hemorrhage, BSCB disruption and neuroinflammation

**Figure 7:**
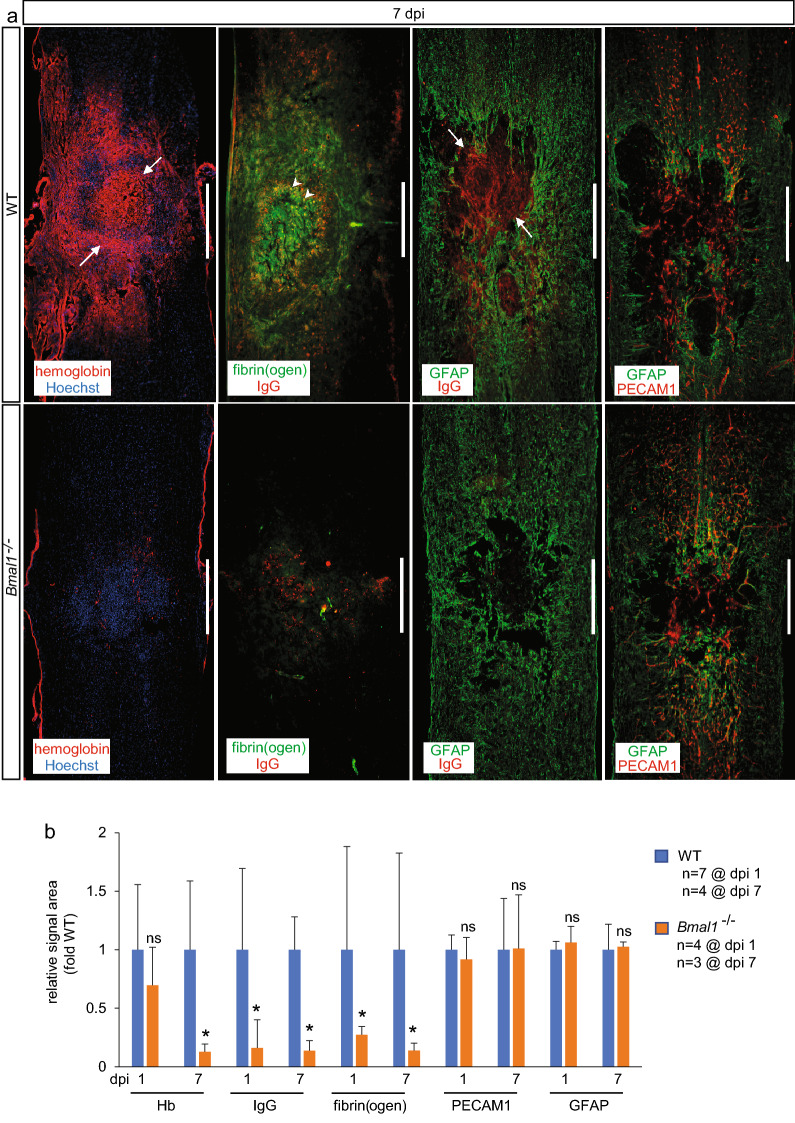
Reduced post-SCI hemorrhage and improved BSCB function in *Bmal1*^*−/−*^ mice. Immunostaining analyses were performed on coronal sections of the spinal cord from WT (dpi 1: M:F = 2:5 including 2 KO littermates; dpi 7: M:F = 2:2) and *Bmal1*^*−/−*^ (dpi 1: M:F = 0:4; dpi 7: M:F = 1:2) mice that received moderate T9 SCI**.** (**a**) In WT mice, signals for the hemorrhage marker hemoglobin (Hb) and extravasated plasma proteins including fibrin/fibrinogen and IgG are strong in the lesion area including the epicenter as indicated by negative staining for the astrocyte marker GFAP or densely packed Hoechst^+^ nuclei of the fibrotic scar cells (arrows). Partial overlap between fibrinogen and IgG signals is also observed (arrowheads). In *Bmal1*^*−/−*^ mice, signals for Hb, fibrin/fibrinogen, and IgG, but not the microvascular marker PECAM1 or GFAP, are reduced. Representative images of the dpi 1 staining are shown in Supplementary Fig. S7. (**b**) Quantification of relative signal area for Hb, IgG, fibrin/fibrinogen, PECAM1 and GFAP in the lesion core (1,500 μm region centered on the injury epicenter and also including the injury penumbra) confirmed reduced subacute hemorrhage, and both acute and subacute BSCB disruption with unaffected content of spinal cord microvasculature or reactive astroglia. Data are means ± SD (*p < 0.05, *u*-test). Similar patterns were observed for another plasma protein, IgM (Supplementary Fig. S8). IgG was detected using an anti-mouse IgG F(ab’)2 fragment to avoid binding to cells that express IgG receptors. Calibration bar is 500 μm.

To confirm pro-BSCB and anti-inflammatory effects of *Bmal1* deletion, markers of hemorrhage, BSCB disruption and neuroinflammation were analyzed by immunostaining. Consistent with intraparenchymal hemorrhage, hemoglobin (Hb) was abundant in the SCI lesion core (or heterodomain^50^) of WT mice as defined by a 1,500 μm region centered on the injury epicenter and including the penumbra (Fig. 7a and Supplementary Fig. S7). On dpi 1, most Hb was found in structures resembling single and/or clustered erythrocytes (Supplementary Fig. S7). On dpi 7, strong Hb signal was observed in and around the injury epicenter as defined by densely packed cells of the fibrotic scar (Fig. 7a). At least partial Hb overlap with such epicenter cells was also apparent (Fig. 7a). That changing pattern likely reflects microglia/macrophage-mediated erythrocyte phagocytosis during hematoma resolution^51^. The Hb signal in *Bmal1*^*−/−*^ mice was reduced at dpi 7, but not dpi 1, suggesting attenuation of the subacute hemorrhage (Fig. 7b).

Tissue deposition of plasma proteins including immunoglobulins and fibrinogen/fibrin is a sensitive marker of SCI-associated BBB/BSCB failure ^10,52,53^. Consistent with those reports, IgG and fibrinogen/fibrin were detected in the SCI lesion core of WT mice on both dpi 1 and dpi 7 (Fig. 7a and Supplementary Fig. S7). In *Bmal1*^*−/−*^ mice, plasma protein deposition was reduced by 80–90% at both time points (Fig. 7b). Similar reductions were also observed for another extravasated protein, IgM (Supplementary Fig. S8). In contrast, the EC cell marker PECAM1, which can reveal both acute post-SCI loss- and later replacement of microvasculature^50,54^, was expressed at similar levels at both time points (Fig. 7 and Supplementary Fig. S7). Likewise, no changes were observed for the reactive astrogliosis marker GFAP (Fig. 7 and Supplementary Fig. S7). These data suggest that in *Bmal1*^*−/−*^ mice, reduced post-SCI extravasation of plasma proteins and decreased hemorrhage are not associated with differences in spinal cord microvascularization or reactive astrogliosis. Therefore, *Bmal1* loss may improve the pro-homestatic potential of ECs and reduce EC/PC dysfunction that results in BSCB failure after SCI.

**Figure 8:**
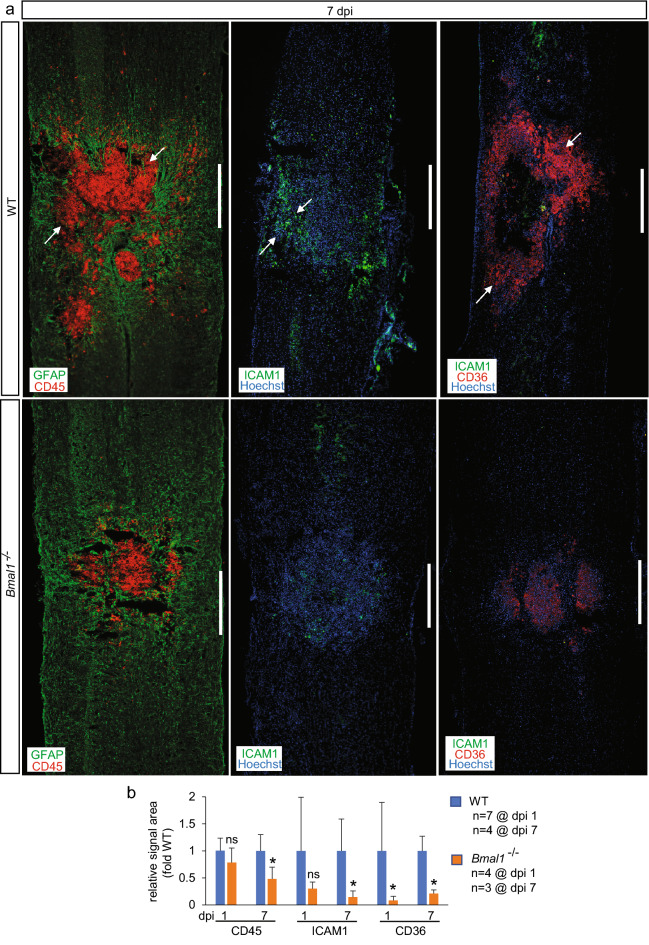
Attenuated post-SCI neuroinflammation in *Bmal1*^*−/−*^ mice. Mice and the immunostaining analyses were as described for Fig. 7. (**a**) Representative images of the lesion area stained for inflammatory markers including the pan-leukocyte membrane protein CD45, the EC/PC/leukocyte cell adhesion molecule ICAM1 and the activated macrophage receptor CD36. Note strong signals in the WT fibrotic scar of the injury epicenter (as defined by lack of GFAP^+^ astrocytes or dense packing of Hoechst^+^ nuclei, arrows). In *Bmal1*^*−/−*^ spinal cords, signals for all three inflammatory markers are reduced. Representative images of dpi 1 staining are shown in Supplementary Fig. S9. (**b**), Quantification of relative signal area for inflammatory markers was performed in the lesion core as described for Fig. 7. Data in (**b**) are means ± SD (*p < 0.05; ns, p > 0.05 *u*-test; at dpi 1, a decreasing trend was observed for ICAM1, p = 0.059); calibration bar is 500 μm.

In SCI lesion area, time-dependent effects on myeloid cell infiltration were also observed (Fig. 8 and Supplementary Fig. S9). Immunostaining for the myeloid cell marker CD45 revealed the presence of those cells scattered throughout the lesion area or concentrated in the GFAP^-^ injury epicenter/fibrotic scar region at dpi 1 or dpi 7, respectively (Supplementary Fig. S9 and Fig. 8a). On dpi 7, but not dpi 1, the relative CD45 signal area was significantly reduced in *Bmal1*^*−/−*^ mice (Fig. 8b). Such time-dependent effects may reflect differential modulation of specific myeloid cell subpopulations including neutrophils or monocyte-derived macrophages that dominate the SCI lesion at dpi 1 or dpi 3–7, respectively^6^.

The pro-inflammatory cell adhesion molecule CD54/ICAM1 was detected in PECAM1^+^ microvessels or densely packed fibrotic scar-like cells of the WT lesion area on dpi 1 or dpi 7, respectively (Supplementary Fig. S9 and Fig. 8a). Those observations are consistent with the reported inflammation-associated ICAM1 expression in ECs, PCs, and macrophages and recognized contributions by the two latter cell types to the fibrotic scar^6,55,56^. Expression of ICAM1 was reduced in *Bmal1*^*−/−*^ mice on dpi 7 (Fig. 8b). A downregulation trend was also observed at dpi 1 (Fig. 8b, p = 0.059). Hence, reduced expression of ICAM1 may reflect decreased pro-inflammatory activity of various types of cells that are present in the lesion area.

CD36 is a pro-inflammatory, poly-valent receptor that is upregulated in macrophages of the fibrotic scar and whose deletion improves post-SCI neuroinflammation, microvasculature function, WMS and functional recovery^42,54^. In WT mice, the pattern of CD36 staining resembled that of CD45 with a scattered signal throughout the lesion area on dpi 1 and its later association with densely packed cells of the fibrotic scar region on dpi 7 (Supplementary Fig. S9, Fig. 8a). In *Bmal1*^*−/−*^ mice, CD36 levels were reduced by at least 80% at both timepoints (Supplementary Fig. S9, Fig. 8b). Therefore, *Bmal1* deficiency not only lowers post-SCI macrophage accumulation, but may also reduce their pro-inflammatory potential.

## Discussion

Consistent with the possibility that SCI may cause local perturbations of the clock pathway to promote secondary injury, we observed acute dysregulation of BMAL1 in the contused spinal cord tissue. However, contrary to our expectations that *Bmal1* KO would worsen outcome after SCI, improved functional recovery, greater WMS and reduced chronic OL loss were observed. Those effects were associated with a remarkable improvement in BSCB function as well as reduced subacute bleeding and neuroinflammation.

The beneficial effects of *Bmal1* deletion in a contusive SCI model were unexpected. Previously, various lines of *Bmal1*^*−/−*^ animals showed spontaneous pathogenic changes in the CNS and/or worsened outcomes following acute neurotoxic injury or autoimmune neuroinflammation. Thus, aging-associated reactive astrogliosis, reduced expression of anti-oxidant enzymes, increased oxidative stress, neuroinflammation, and synaptic damage were observed after germ line-, pan-neural-, or astrocyte-selective deletion of *Bmal1*^20,57^. In addition, age-dependent chronic BBB dysfunction was associated with loss of *Bmal1* from nestin^+^ PCs^58^. The *Bmal1*^+*/−*^ genotype enhanced acute striatal neurotoxicity of 3-nitropropionic acid^20^. In monocyte-selective *Bmal1*^*−/−*^ mice, EAE resulted in enhanced locomotor deficits, increased neuroinflammation and demyelination in the spinal cord^26^. Hence, the critical question emerges as to why does germline *Bmal1* deletion reduce SCI-associated pathology even though it involves pathogenic cascades that are negatively regulated by BMAL1 in other models? One explanation of this apparent paradox could be that BMAL1 plays a role in upstream events that initiate the critical effector components of the secondary injury including oxidative stress, neuroinflammation, OL loss, axonal damage, and subsequent tissue scarring.

What could such a clock pathway-modulated trigger for pathogenic events in SCI be? SCI-induced intraparenchymal bleeding, activation of the hemostasis pathway and BSCB disruption lead to intraspinal deposition of hemoglobin, iron, fibrinogen/fibrin, immunoglobulins and complement components that, in turn, promote oxidative stress, death of neurons and OLs, neuroinflammation, WM injury, scarring, and de-novo formation of leaky/dysfunctional blood vessels^3,9,50,53,59–62^. Axonal injury, impaired OL replacement and reduced axonal growth are additional potential consequences of blood extravasation/BSCB dysfunction in SCI^10,63^. Therefore, genetic or pharmacological manipulations that promote hemostasis and maintenance of the BSCB reduce downstream pathologies in SCI including neuroinflammation, tissue loss and locomotor impairment^60,64,65^. Extensive effects on the EC/PC transcriptome that are consistent with reduced subacute hemorrhage and BSCB preservation support a critical role of the vascular compartment in improving SCI outcome in *Bmal1*^*−/−*^ mice. Those anti-hemorrhagic/pro-BSCB effects may outweigh potential negative consequences of *Bmal1* loss on anti-oxidant defenses and/or neuroinflammation.

How could *Bmal1* deficiency improve post-SCI hemostasis and/or BSCB integrity? Interestingly, germ-line *Bmal1*^*−/−*^ mice show an age-dependent increase of pro-thrombotic potential associated with: (i) elevated levels of the coagulation factors fibrinogen, F7, F8 and VWF, (ii) higher platelet count, (iii) increased platelet aggregation and adhesion to ECs, and, (iv) reduced EC expression of anti-thrombotic molecules including a candidate clock pathway target gene thrombomodulin (*Thbd*)^29^. Loss of BMAL1-mediated transcriptional regulation in the liver and ECs was proposed to mediate increases in fibrinogen, F7 and F8, respectively^29^. EC-specific effects including reduced activity of eNOS/NOS3 or expression and/or secretion of VWF were implicated in altered platelet function^28,29^. We did not observe any mRNA changes of the coagulation factors or platelet counts between WT or *Bmal1*^*−/−*^ animals with SCI as determined in livers or peripheral blood at dpi 1, respectively (Supplementary Table S3). The younger age of our experimental animals (8–10 weeks) and/or the acute systemic effects of SCI may explain why our findings differ from published reports. Indeed, increased platelet counts and F8 levels were only observed in 30 week old naïve *Bmal1*^*−/−*^ mice^29^. Moreover, as an established acute phase protein, fibrinogen may be induced after SCI nullifying any baseline differences. However, in the contused cord tissue, *Bmal1* loss reduced levels of *Nos3*/*eNos*, *Aplnr* and *Ptgis*, suggesting increased pro-thrombotic potential of *Bmal1*^*−/−*^ ECs.

Reduction of NOS3 expression/activity could also explain improved BSCB function as NOS3-derived NO has been implicated in pathological disruption of the BBB^66^. Importantly, *Bmal1* deletion reduced the expression of one third of the genes that are co-upregulated during BBB/BSCB disruption^44^. Some *Bmal1* KO-reduced components of the BSCB disruption module such as those related to ECM deposition or EC proliferation/angiogenesis may represent an attempt to restore BSCB. Several others including *Ptgs2*, *Adamts8*, and, *Ccl2* are likely contributors to BSCB failure. As neoangiogenesis plays a role in delayed BSCB failure after SCI^50^, *Bmal1*^*−/−*^ mediated downregulation of pro-angiogenic genes of the BBB/BSCB module could be another mechanism to reduce delayed BSCB dysfunction after SCI. However, as the post-SCI content of microvasculature appeared similar in WT and *Bmal1*^*−/−*^ mice, slowing down rather than blocking post-SCI angiogenesis could be a contributing factor to long-term BSCB improvement.

The positive effects of *Bmal1* deletion on post-SCI hemostasis and BSCB function are likely mediated by its activity in ECs and PCs. Supporting this contention, a pro-thrombotic phenotype has been reported after EC-selective deletion of *Bmal1*^27^. Moreover, reduced eNOS3 activity and increased platelet adhesion appeared to occur in *Bmal1*^*−/−*^ ECs in a cell autonomous manner^28,30^. Additionally, we confirmed *Bmal1* expression in spinal cord ECs. Lastly, EC/PC-specific transcriptomes were modified in *Bmal1*^*−/−*^ mice including downregulation of many genes involved in BSCB disruption. Therefore, loss of *Bmal1* in vascular cells may be the primary driver of improved SCI outcome in *Bmal1*^*−/−*^ mice outweighing any pro-pathogenic effects on neurons, OLs, OPCs, macrophages, and/or microglia.

Current pro-BSCB effects of the germ-line *Bmal1* deletion after SCI might at first appear inconsistent with a report of chronic BBB impairment after *Bmal1* deletion in Nes^+^ cells, including PCs^58^. The latter phenotype was associated with reduced PC coverage of the brain microvasculature but not PC loss. While PC association with microvasculature contributes to development and maintenance of a functional BBB under physiological conditions, PCs may also be involved in BBB breakdown after injury^56,67^. Therefore, it is tempting to speculate that while *Bmal1* KO perturbs the physiological function(s) of PCs to stabilize BBB/BSCB, it also attenuates their pro-inflammatory/anti-BBB activity in response to injury. At least after contusive SCI, the net effects would be improved BSCB function and reduced neuroinflammation. Noteworthy, BBB/BSCB formation and repair are also promoted by astrocytes^46^. As in *Bmal1*^*−/−*^ mice, the response of astrocytes to SCI appears to change, the BSCB improvement may also have an astrocytic component.

Our findings that BMAL1 may contribute to SCI by promoting hemorrhage and BSCB disruption and thereby enhance neuroinflammation and OL loss raise an important question. Are those key components of SCI pathogenesis subject to circadian regulation by the clock pathway? Although there are no data in the literature on injury time of day effects on SCI pathology and post-SCI recovery, such effects were reported in vascular CNS disease models including ischemic stroke or subarachnoid hemorrhage^68,69^. In those cases, outcome worsened when injuries occurred at early circadian times with maximal BMAL1 expression^68–70^. Conversely, opposite time of day effects were reported in sepsis models, where loss of anti-inflammatory activity of BMAL1 worsens outcome ^22–24^. Interestingly, at least some aspects of the BSCB/BBB function show circadian oscillations and may be regulated by the clock pathway. That includes the ability of plasma TNFα to penetrate into the spinal cord, which peaks at the beginning of the day when BMAL1 expression is high^70,71^. Of note, BSCB permeability for TNFα is compromised after SCI for up to dpi 5^72^. Hemostasis is also under BMAL1-dependent circadian control^28,73^. Therefore, circadian oscillations in the function of those systems may contribute to injury time of day effects in neurotrauma. The extent of such contributions may vary depending on the type and severity of the injury. Future experiments are needed to test such effects in the context of contusive SCI. Lastly, as the spinal cord clock pathway is transiently disrupted by thoracic SCI^74^ (Fig. 1), one could ask whether such a clock dysfunction may help to restore BSCB and/or decrease bleeding.

Collectively, our findings reveal an unexpected role of BMAL1 in BSCB disruption and intraspinal bleeding after thoracic contusive SCI. They also raise the question whether similar mechanisms may contribute to other BBB/BSCB-disruptive and/or bleeding-associated CNS pathologies such as stroke or TBI^46^. Indeed, a reduced proliferative response of glia and sex-dependent neuroprotection were reported in *Bmal1*^*−/−*^ mice after a photothrombotic cortical stroke^75^. However, given potentially pleiotropic and mutually opposing effects of BMAL1 on the pathogenesis of acute CNS injury, testing such an interesting possibility would require unequivocal identification of cells in which loss of *Bmal1* results in neuroprotection and improved recovery.

## Materials and methods

### Animals

*Bmal1*^+/−^ (*Arntl*^*tm1Bra*^, stock #009100, C57Bl/6J background) and WT C57Bl/6 J mice were obtained from the Jackson Laboratory (Bar Harbor, ME). *Bmal1*^*−/−*^ mice were obtained from crosses of *Bmal1*^+/−^ animals as *Bmal1*^*−/−*^ mice are sterile. Age- and sex-matched WT control mice were ordered from the Jackson Laboratory and acclimated for at least two weeks before the start of a study. Whenever possible, WT littermate controls were used as indicated. All reported animal experiments were approved by the University of Louisville Institutional Animal Care and Use Committee and Institutional Biosafety Committee and strictly adhered to NIH guidelines on use of experimental animals. Additional information on experimental design and analysis is provided in Supplementary Methods including the Supplementary Table S2 that summarizes animal use information.

### Materials

Antibodies were from commercial suppliers as indicated (Supplementary Table S4). All other reagents and materials were purchased from Sigma Aldrich (St. Louis, MO), VWR International (Radnor, PA), Thermo Fisher Scientific (Waltham, MA), or Integrated DNA Technologies (IDT, Coralville, IA).

### Spinal cord injury

*Spinal cord injury* was performed as described previously^76^. Briefly, 8–10 week old animals were anaesthetized by an i.p. injection of 400 mg/kg body weight 2,2,2-tribromoethanol. Lacri-Lube ophthalmic ointment (Allergen, Irvine, CA) was applied to prevent drying of eyes. Moderate contusion injuries (50 kdyn force/400–600 μm displacement) were performed at the T9 level using the IH impactor (Infinite Horizons, Lexington, KY) following a laminectomy at T9 vertebrae. Postoperative care included s.c. 0.1 ml saline (immediately after surgery), 5 mg/kg s.c. gentamicin every 24 h for 7 days, 0.1 mg/kg s.c. buprenorphine every 12 h for 2 days, and manual expression of bladders twice a day for seven to ten days or until spontaneous voiding returned. All surgical and post-operation procedures were completed according to NIH and IACUC guidelines.

### Hindlimb locomotor function

*Hindlimb locomotor function* was evaluated in an open field using the Basso Mouse Scale (BMS)^37^. Evaluations were performed at baseline before the injury and weekly thereafter. All raters were trained by Dr. Basso and colleagues at the Ohio State University and were blinded to genotype or surgical identity.

### Tissue collection and processing

To avoid time-dependent fluctuations of BMAL1 expression^16,70^, all tissue collections were performed at 9–11 a.m. (ZT 3–5) except 6 h sham/SCI samples for RNA analysis that were collected at ZT 8–9 (Fig. 1). Mice were deeply anaesthetized using 2,2,2-tribromoethanol and perfused transcardially with ice cold phosphate buffered saline (PBS, 30 ml/mouse). For RNA analysis, spinal cord samples (5 mm centered on the injury epicenter) were collected and frozen in liquid nitrogen. For histology, mice were perfused with 4% paraformaldehyde (PFA, pH 7.4 in PBS). Dissected spinal cords were post-fixed (4% PFA for 1 h at 4 °C), cryoprotected in 30% sucrose (pH 7.4, 72 h at 4 °C) and mounted in TFM tissue freezing medium (GeneralData, Cincinnati, OH). Cryostat sections (20 μm) were cut, thaw mounted on slides and stored at − 20 °C until further use.

### qPCR analysis

RNA was extracted using Trizol or RNeasy Lipid Tissue Mini Kit (Qiagen, Germantown, MD) and cDNA was synthesized following standard methodology. SYBR Green master mix- or Taqman DNA polymerase and ViiA 7 Real-Time PCR System (Applied Biosystems, Grand Island, NY) were used for non-labeled or Taqman primers, respectively (see Supplementary Table S5 for primer information). The delta delta CT method was used for quantification; the normalizing transcript was *Gapdh*.

### RNASeq analysis

Total RNA was extracted using RNeasy Lipid Tissue Minikit (Qiagen #74804) and poly-A-enriched libraries were prepared and sequenced using Illumina NextSeq 500. Number of raw reads/sample was 50,185,248 to 57,727,458 (average 52,643,643). At least 97.6% reads were aligned to the mouse reference genome (average read alignment 98.1%). DESeq2 was used to identify differentially expressed genes (q-value < 0.05). The raw counts were normalized using DESeq2’s default method, relative log expression (RLE). See Supplementary Methods for additional details. The RNA-seq data generated in this study were deposited in the Gene Expression Omnibus (GSE151685).

### Publicly available transcriptome data sets

The top 500 microglia, astrocyte, OPC, OL or neuron-enriched transcripts that were identified in mouse brains at postnatal days 7 or 17 were downloaded from the brain RNASeq database (www.brainrnaseq.org)^43^. The top 1,000 expressed genes (as defined by fpkm values) in mouse spinal cord macrophages on dpi 3 after contusive SCI were accessed from GSE84737^42^. The brain EC-enriched genes were defined based on data that were obtained from genetically labeled mouse brain ECs of young adult mice^44^. The list included 467 EC-enriched genes with log2FC values > 1.5 (q < 0.05) when comparing to all vascular cell or total brain mRNAs, respectively. The 260 brain pericyte (PC)-enriched genes were defined based on appearance in at least 2 of 5 different mouse brain mural transcriptome data sets^45^. The BBB/BSCB disruption module of the EC-enriched transcriptome included 136 genes that were upregulated in at least 3 of 4 acute injury models at subacute timepoints, when the BBB/BSCB dysfunction was maximal (dpi 3 in TBI and the MCAO model of stroke, dpi 2 in the kainic acid model of status epilepticus, and 1 day after leveling off clinical disease score in the EAE model)^44^.

### Gene ontology (GO) analysis

NCBI’s DAVID was used to perform gene ontology enrichment analysis. All 17,207 transcripts detected by RNASeq in spinal cord samples were used as a background and FDR q < 0.1 was used as a cut off. GO categories analyzed included Biological Process (BP), Cellular Component (CC) and Molecular Function (MF) as indicated.

### WMS analysis

WMS was evaluated using eriochrome cyanine (EC) staining for myelin as described previously^76^. A detailed description is provided in Supplementary Methods.

### Immunostaining

Slides with mounted spinal cord sections were warmed at 37 °C for 10 min, washed 3 × with PBS, and blocked in TBS + 0.1% Triton X-100, 0.5% BSA, and 10% normal donkey or goat serum for 1 h at room temperature and then incubated overnight at 4 °C with primary antibodies in blocking buffer, followed by 3 × washes and incubation in secondary antibodies at 25 °C for 1 h. Negative controls included appropriate species-specific non-immune Ig subtypes instead of primary antibodies. For BMAL1 immunostaining, sections were initially permeabilized in 0.5% NP-40 for 10 min before applying blocking reagent (10% normal goat serum); the primary antibody (or antibodies in case of co-staining) were applied in 0.1% Triton X-100 and 5% normal goat serum for 2 h at room temperature. The list of primary and secondary antibodies is presented in Table S4. Some sections were additionally counter-stained with Hoechst-33258. Negative controls for each antibody were done by parallel substitution of species-matched pre-immune IgG and resulted in no staining (Supplementary Fig. S1).

*Imaging.* Images were captured with Zeiss Observer.Z1 fluorescent microscope using a 10 × or 40 × objective or Nikon Eclipse Ti inverted microscope equipped with a 4 × objective. Identical exposure times were used for corresponding injured and control spinal cords. Digitized images were captured using AxioVision (Zeiss) or Elements software (Nikon Instruments) followed by conversion to TIFF files. All images used for quantitative analyses were acquired and analyzed without knowledge of animal group assignment.

### OL content and axonal density analysis

Quantification of fluorescent immunostained images followed previously described methodology with minor modifications using transverse 20-μm-thick sections that were co-stained with CC1 (OL marker) and anti-NFH (axonal marker) antibodies^38^ (see Supplemental Methods for more details).

### Hemorrhage-, BSCB disruption-, and, neuroinflammation marker analysis

Every fifth coronal section from each cord (five 20 μm sections/animal) was immunostained for the indicated markers and photographed with a 10 × objective and stitched together using Elements software during acquisition. Elements software was used to threshold baseline brightness and contrast identically for each image for all quantitative object and field measurements. The lesion site was defined as 1,500 μm region that was centered in the injury epicenter and included the injury penumbra. It corresponds to a heterodomain that is a pathology-affected region exhibiting extravascular deposition and disorganization of vascular laminin as well as disrupted GFAP immunoreactivity^50,54^. To assess the presence of hemorrhage, BSCB disruption, or inflammatory markers, the total area within the lesion site positive for each marker was quantified by digital image analysis using the basic densitometric thresholding features of Elements software, similar to methods previously reported^54^. Threshold values were obtained and set for each marker and held constant for each image quantified. The percentage of the lesion site area positive for each marker was quantified. For each animal, at least 3 sections spanning the injury epicenter were analyzed and marker signal area was averaged and normalized to WT control values.

### Statistical analysis

Repeated measures ANOVA (RM-ANOVA) followed by Tukey post hoc tests was used for analyzing BMS locomotor recovery data and chronic effects on OL cell content and axonal density. All other qPCR or image analysis data were analyzed using the non-parametric Mann–Whitney test (*u*-test, single sided).

## Supplementary information

Supplementary Information.

Supplementary Table.
