# Supplementary material for: Improved locomotor recovery after contusive spinal cord injury in Bmal1−/− mice is associated with protection of the blood spinal cord barrier
Source: Sci Rep. 2020 Aug 26;10:14212. doi: 10.1038/s41598-020-71131-6 (PMC7450087; doi:10.1038/s41598-020-71131-6)
Supplement: Supplementary file 1 — Supplementary Information. [file 41598_2020_71131_MOESM1_ESM.pdf]

Improved locomotor recovery after contusive spinal cord injury in *Bmal1*$^{-/-}$ mice is associated with protection of the blood spinal cord barrier.

Lukasz P. Slomnicki, Scott A. Myers, Sujata Saraswat-Ohri, Molly V. Parsh, Kariena R. Andres, Julia H. Chariker, Eric C. Rouchka, Scott R. Whittemore and Michal Hetman

<u>**Supplementary Information**</u>

Supplementary Methods

Supplementary References

Supplementary Figures S1-S10

Supplementary Tables S1-S5 (Tables S2-S5 are part of this document, Tables S1 is supplied as a separate MS Excel spreadsheet)

Supplementary Methods

Experimental design: sample size determination. For analysis of locomotor recovery, *a priori* power calculations were performed. From BBB data on hindlimb function recovery in rats that were collected at Louisville over the past 27 years, power analyses show the ability to detect a significant difference is >95% with a sample size of 10/group, based on the observed standard deviations between 1.2-1.9 and an effect size of 10% (*e.g.* difference between group scores). As the variance of BBB and BMS evaluations are similar [1,2] , functional analyses using n=10 *Bmal1*$^{-/-}$ and n=14 WT mice were adequately powered to detect at least 10% change in BMS (99.9% power to detect the observed BMS difference of 1.59 at $p<0.05$). For all other studies, no statistical tests were used to predetermine sample size. Instead, sample sizes were rationalized by considering sufficient replication (weighing the level of biological variation) as well as censoring due to inadvertent losses of animals or samples. In most of those cases a priori design was not possible due to lack of reliable prior data to define variability.

Experimental design: gender. In studies with WT mice (Figs. 1-3), females were used. Females are predominantly used in SCI literature due to lower incidence of urinary tract infections and, therefore, have better survival as compared to males. Thus, use of females helps to reduce the overall animal number needed for SCI experiments. As *Bmal1*$^{-/-}$ mice are sterile, those animals have to be generated by breeding heterozygous parents thereby limiting availability of suitable experimental subjects. Therefore, to properly power experiments that involved *Bmal1*$^{-/-}$ mice (Figs. 4-8) we used both sexes. Such a strategy is justified by a documented lack of significant sex effects on locomotor recovery, neuroinflammation or lesion volume after contusive injury in C567Bl6 mice [3]. However, sex unbalanced groups emerged in our studies on *Bmal1*$^{-/-}$ mice due to (i) limited availability of males of comparable age, (ii) peri-operative loss of animals, (iii)

initial genotyping errors that were later corrected by terminal genotyping and resulted in group re-assignments.

Group allocation and blinding. Group allocation was not random. Instead, biological controls were used in all experiments. Such a strategy was necessary due to limited number of *Bmal1*$^{-/-}$ animals and the need to pair them with comparable WT control animals including similar age, sex and, if possible, same origin. For each study surgeries were performed on the same day or two consecutive days (ZT3-ZT5) with random order of animals and without knowledge of group assignment. Likewise, all behavioral and histological analyses were blinded.

RNASeq analysis. Spinal cord tissue that was isolated from *Bmal1*$^{-/-}$ (n=3, 2 females, 1 male) and WT mice (n=4, 2 females, 2 males) was homogenized on ice with Tissue-Tearor (BioSpec Products, Bartlesville, OK). Total RNA was extracted using RNeasy Lipid Tissue Minikit (Qiagen #74804) according to the manufacturer's instructions. The quality of RNA was accessed by capillary electrophoresis using an Agilent Bioanalyzer. One microgram of total RNA was used for poly-A enrichment. First and second cDNA strands were synthesized followed by 3' end adenylation. Libraries were prepared using the TruSeq Stranded mRNA Library Prep Kit along with TruSeq RNA Index Set A according to manufacturer's instructions (Illumina, San Diego, CA). Samples were barcoded with Illumina TruSeq Adapters, DNA fragments were enriched by PCR reaction and their quality was validated on an Agilent Bioanalyzer. All 1.8 pM libraries were then denatured and sequencing was performed at the University of Louisville Genomics Core Facility Illumina NextSeq 500 using the NextSeq 500/550 75 cycle High Output Kit v2.5 (Illumina, Carlsbad, CA). Raw read number ranged from 50,185,248 to 57,727,458 with an average of 52,643,643 reads/sample. The quality control of the raw sequence data was performed using FastQC v.0.10.1 and the sequences were directly aligned to the *Mus musculus*

reference genome assembly (GRCm38.p6.fa) using Tophat 2v.2.0.13. At least 97.6% reads were aligned to the mouse reference genome (average read alignment was 98.1%). For the DESeq2 analysis of differential gene expression raw counts were obtained from the Tophat aligned bam format files using HTSeq v.0.10.0. The raw counts were normalized using DESeq2's default method, relative log expression (RLE). A q-value < 0.05 was used as a criterion for defining differentially expressed genes.

WMS analysis. WMS was evaluated as described previously [4,5]. Briefly, serial transverse sections spanning -1 mm rostral to +1 mm caudal from the lesion epicenter were stained for myelin using iron eriochrome cyanine (EC) with an alkali differentiator modification. Images were captured with a 4x objective on a Nikon Eclipse Ti inverted microscope. Myelinated, EC^+ WM was traced using Nikon Elements software. The injury epicenter was identified based on the least relative content of WM as defined by EC^+ area/total section area. Percent sparing was calculated after normalizing the data to average EC^+ WM content in naïve female C57BL6/J mice (n=4, T8-T10 level, 8-10 week old). All imaging and analysis was performed without knowledge of genotype or treatment.

OL content and axonal density analysis. The procedure followed previously described methodology with minor modifications [6]. Briefly, transverse 20 μm sections were co-stained with the CC1 (OL marker) and anti-NFH (axonal marker) antibodies. Nuclei were counter-stained with Hoechst-33258. The sections were selected based on results of WMS analysis that was done on a set of adjacent sections to define the injury site. Following image capture (Zeiss Observer.Z1, 10x objective, identical exposures) digitalized pictures were saved as gray scale jpeg files. A grid of 0.01 mm^2 squares (100 μm x 100 μm) was overlaid on the images over the ventral/ventrolateral WM. The numbers of $CC1^+$ OLs and all cells (defined by Hoechst-stained

nuclei) were counted manually in ventral (V) and ventrolateral (VL) WM on each side at the injury epicenter and -1 mm rostrally or +1 mm caudally. At -1 mm or +1 mm V WM was defined as WM located between the tips of the ventral horns and VL WM was defined as WM lateral to the ventral horns and ventral to a coronal plane line through the middle point of the central canal. In the epicenter region, V WM was defined as an area between transverse or sagittal plane lines that were tangential to ventral or lateral borders of the grey matter lesion, respectively. The epicenter VL WM was located between the coronal and sagittal plane lines that intersected the middle point of a section and were tangential to a lateral border of the grey matter injury site, respectively. The grey matter injury site was identified as an area that contained densely packed cells of the fibrotic scar, as visualized with Hoechst. For each animal, two sections from each region were analyzed; in each section all cells in the V- or VL WM were counted. The average total number of cells counted per animal was 466 or 426 or 502 for V WM and 692 or 629 or 738 for VL WM at the epicenter, -1 mm, or, +1 mm locations, respectively. Axonal density was quantified using the same sections and identical region definitions as described for cell counting. Each region was traced on both sides and the areas of the positive NFH signal and Hoechst-stained nuclei were determined. The area measurements were performed using the NIH ImageJ after adjusting the “threshold” parameter to separate a positive signal from the background. The threshold parameter was manually adjusted for each section to cover exclusively positive signals of NFH or Hoechst. The ratio of pixels above the threshold to all pixels was calculated for both NFH and Hoechst and then presented as the NFH/Hoechst ratio (axonal density). The imaging, as well as image processing and analysis including cell counting, were done without knowledge of the genotype.

Supplementary References

1. Engesser-Cesar, C., Anderson, A. J., Basso, D. M., Edgerton, V. R. & Cotman, C. W. Voluntary wheel running improves recovery from a moderate spinal cord injury. *J Neurotrauma* **22**, 157-171, doi:10.1089/neu.2005.22.157 (2005).
2. Basso, D. M. *et al.* Basso Mouse Scale for locomotion detects differences in recovery after spinal cord injury in five common mouse strains. *J Neurotrauma* **23**, 635-659, doi:10.1089/neu.2006.23.635 (2006).
3. Luchetti, S. *et al.* Comparison of immunopathology and locomotor recovery in C57BL/6, BUB/BnJ, and NOD-SCID mice after contusion spinal cord injury. *J Neurotrauma* **27**, 411-421, doi:10.1089/neu.2009.0930 (2010).
4. Magnuson, D. S. *et al.* Functional consequences of lumbar spinal cord contusion injuries in the adult rat. *J Neurotrauma* **22**, 529-543, doi:10.1089/neu.2005.22.529 (2005).
5. Ohri, S. S. *et al.* Attenuating the endoplasmic reticulum stress response improves functional recovery after spinal cord injury. *Glia* **59**, 1489-1502, doi:10.1002/glia.21191 (2011).
6. Lytle, J. M. & Wrathall, J. R. Glial cell loss, proliferation and replacement in the contused murine spinal cord. *Eur J Neurosci* **25**, 1711-1724, doi:10.1111/j.1460-9568.2007.05390.x (2007).
7. Zhang, Y. *et al.* An RNA-sequencing transcriptome and splicing database of glia, neurons, and vascular cells of the cerebral cortex. *J Neurosci* **34**, 11929-11947, doi:10.1523/JNEUROSCI.1860-14.2014 (2014).

Figure S1. Validation of BMAL1 immunostaining. Staining with the rabbit anti-BMAL1 antibody or normal rabbit non-immune IgG was performed in WT and *Bmal1*$^{-/-}$ spinal cord tissue as indicated. The tissue was prepared at dpi 7 as described in Fig. 7. High power images of cells in the penumbral WM are shown. Note the absence of BMAL1 signal in *Bmal1*$^{-/-}$ tissue. Also, no signal was observed in WT tissue using non-immune IgG instead of anti-BMAL1 antibody.

Figure S2. BMAL1 is ubiquitously expressed in spinal cord neurons. Low power images of co-immunostaining for BMAL1 and the pan-neuronal marker NeuN in WT spinal cord of sham and SCI mice at dpi 1 (animals and sections were as described for Fig. 2; SCI images are from the injury penumbra region, 0.5-1 mm from the injury epicenter). Arrows and arrowheads point to BMAL1^{+} neurons.

Figure S3. BMAL1 is expressed in spinal cord astrocytes. Low power images of co-immunostaining for BMAL1 and the astrocyte marker GFAP in WT spinal cord of sham and SCI mice at dpi 1 (animals and sections were as described for Fig. 2; SCI images are from the injury penumbra region, 0.5-1 mm from the injury epicenter). Arrows and arrowheads point to BMAL1$^+$ astrocytes.

Figure S4. BMAL1 is expressed in spinal cord OLs. Low power images of co-immunostaining for BMAL1 and the OL marker epitope CC1 in WT spinal cord of sham and SCI mice at dpi 1 (animals and sections were as described for Fig. 2; SCI images are from the injury penumbra region, 0.5-1 mm from the injury epicenter). Arrows and arrowheads point to BMAL1$^+$ OLs. In SCI tissue, positive CC1 staining in grey matter neuron-like cells is likely non-specific (asterisks).

Figure S5. BMAL1 is detectable in some spinal cord microvessels. Low power images of co-immunostaining for BMAL1 and the EC marker PECAM1 in WT spinal cord of sham and SCI mice at dpi 1 (animals and sections were as described for Fig. 2; SCI images are from the injury penumbra region, 0.5-1 mm from the injury epicenter). Arrows and arrowheads point to BMAL1$^+$ ECs.

Figure S6. High representation of OPC-enriched genes among those downregulated in *Bmal1*$^{-/-}$ spinal cord may be biased due to extensive reduction in proliferation-associated genes in *Bmal1*$^{-/-}$ mice. ***(a)*** Gene count of the overlap between *Bmal1*$^{-/-}$ highly regulated mRNAs

and the top 500 brain OPC-enriched transcripts that were obtained from the BrainRnaSeq data base [7]. ***(b)*** GO enrichment for *Bmal1*$^{-/-}$ downregulated OPC genes. Note the high enrichment of cell proliferation-associated GOs. ***(c)*** GO enrichment for the top 500 brain OPC-enriched genes (of those 392 were annotated by NCBI's DAVID GO:BP algorithm) reveals high representation of cell proliferation-associated transcripts. As at dpi 3, SCI triggers proliferative responses in various cell types including OPCs, macrophages/microglia, ECs, PCs and perivascular fibroblasts. The apparent enrichment of pro-proliferative "OPC" genes in the *Bmal1*$^{-/-}$-downregulated transcriptome may represent reduced proliferation of those non-OPC cells.

Figure S7. Reduced staining for the hemorrhage/BSCB disruption markers in *Bmal1*$^{-/-}$ mice at dpi 1. The animals used and the subsequent immunostaining analyses were as described for Fig. 7. Representative images of immunostaining signals for hemoglobin (Hb), fibrin/fibrinogen, IgG, PECAM1 ***(a)***, and, GFAP ***(b)*** throughout the lesion site including the injury epicenter and the penumbra. Note absence of GFAP^{+} astrocytes from the injury epicenter (arrows). Relative staining area quantifications revealed reduced levels of fibrin/fibrinogen and IgG but not Hb, PECAM1 or GFAP (Fig. 7b). Calibration bar is 500 μm.

Figure S8. Reduced post-SCI extravasation of IgM in *Bmal1*$^{-/-}$ mice. The animals used and the subsequent immunostaining analyses were as described for Fig. 7. ***(a)*** Representative images of the lesion site. ***(b)*** IgM signal area quantifications. Note reduction of IgM staining in *Bmal1*$^{-/-}$ mice at both dpi 1 and dpi 7. Data in *(b)* are means ± SD (*, $p<0.05$, *u*-test). Calibration bar is 500 μm.

Figure S9. Reduced staining for the inflammatory markers CD36 and ICAM1, but not CD45, in *Bmal1*$^{-/-}$ mice at dpi 1. The animals and immunostaining analysis were as described for Fig. 7. ***(a)*** Representative images of CD45, ICAM1 and CD36 staining in the lesion site. Note the scattered signals for CD45 and CD36 throughout the entire lesion area in WT tissue. At least some WT ICAM1 signal appears vascular. In *Bmal1*$^{-/-}$ samples, the CD45 staining pattern is similar to WT, while ICAM1 and CD36 signals appear reduced. Relative staining area quantifications confirmed significant reduction for CD36 and a downregulation trend for ICAM1 (p=0.059) but not CD45 (Fig. 8b). ***(b)*** Partial microvascular association of ICAM1 is indicated by co-localization with PECAM1 (arrows). Calibration bars are 500 μm *(a)* and 100 μm *(b)*.

Figure S10. The untrimmed image of the western blot shown in Fig. 1b. Positions of the molecular weight markers are indicated. Note presence of major bands with molecular masses corresponding to the expected size of the detected targets (mouse BMAL1, 69.4 kD, mouse GAPDH 35.8 kD).

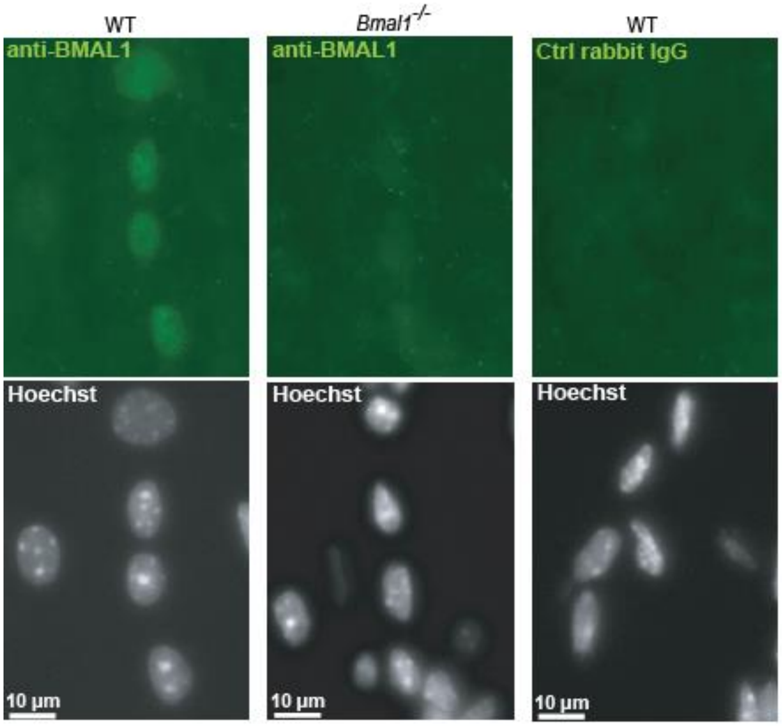

Slomnicki, Myers, Saraswat-Ohri et al., Fig. S1

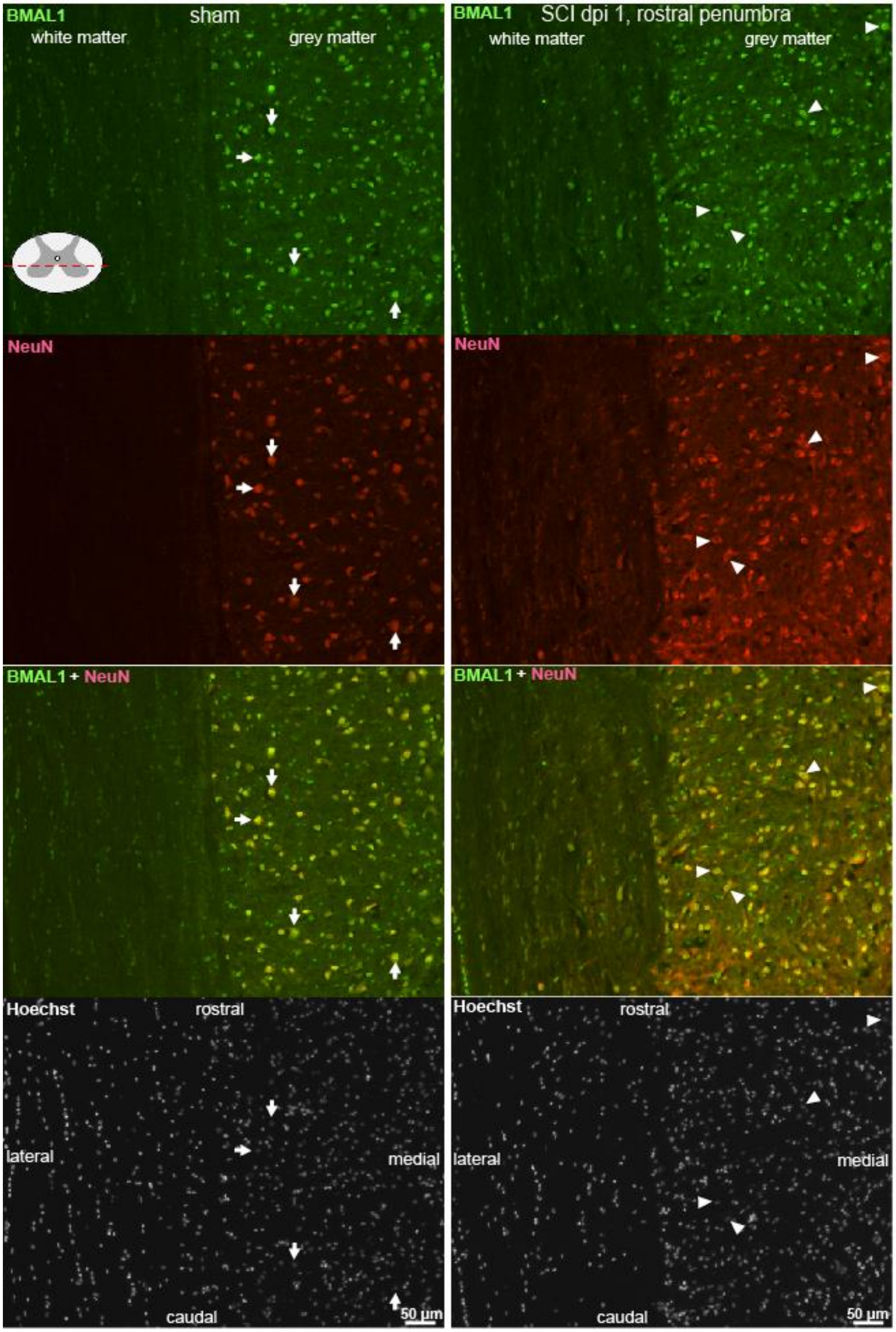

Slomnicki, Myers, Saraswat-Ohri et al., Fig. S2

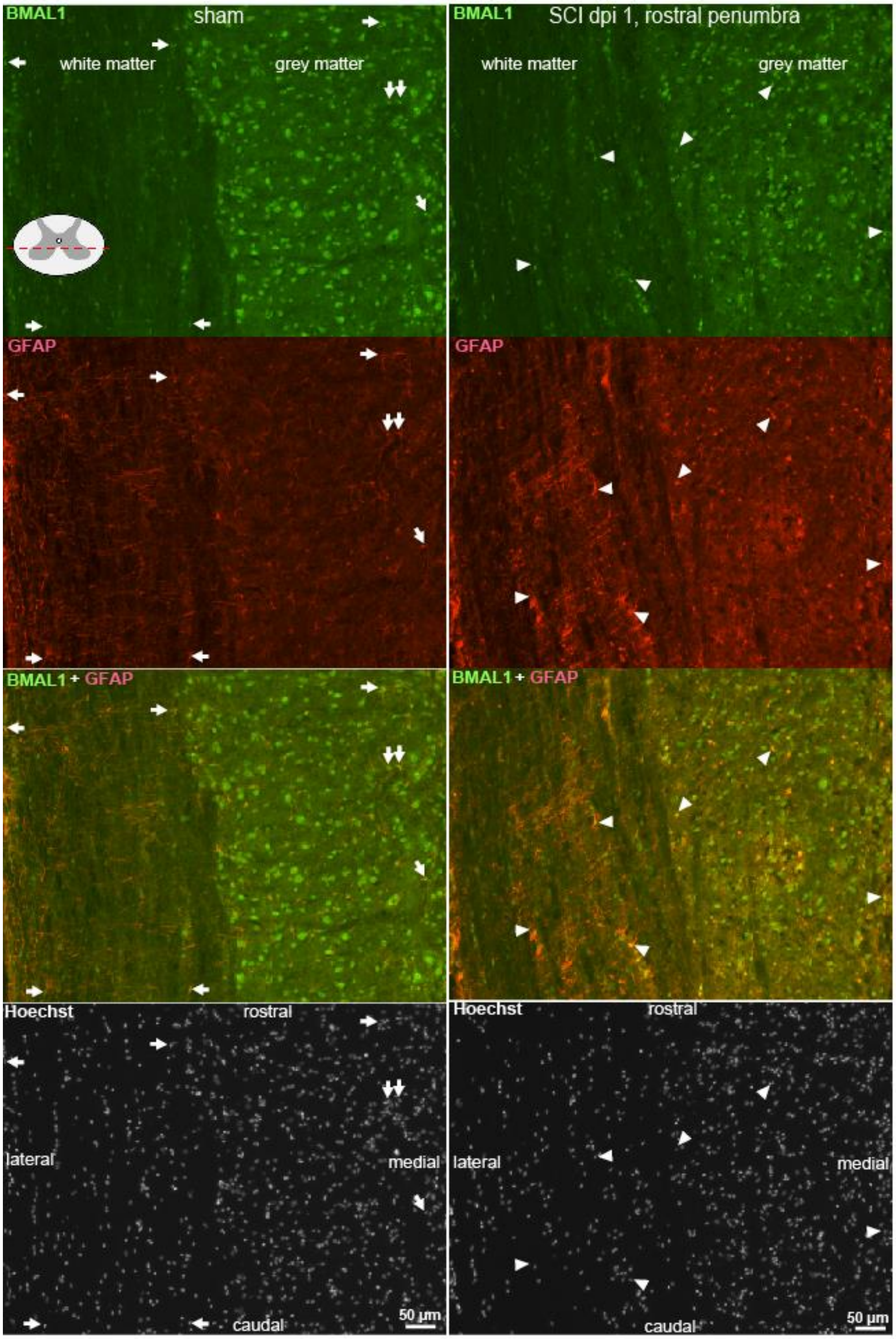

Slomnicki, Myers, Saraswat-Ohri et al., Fig. S3

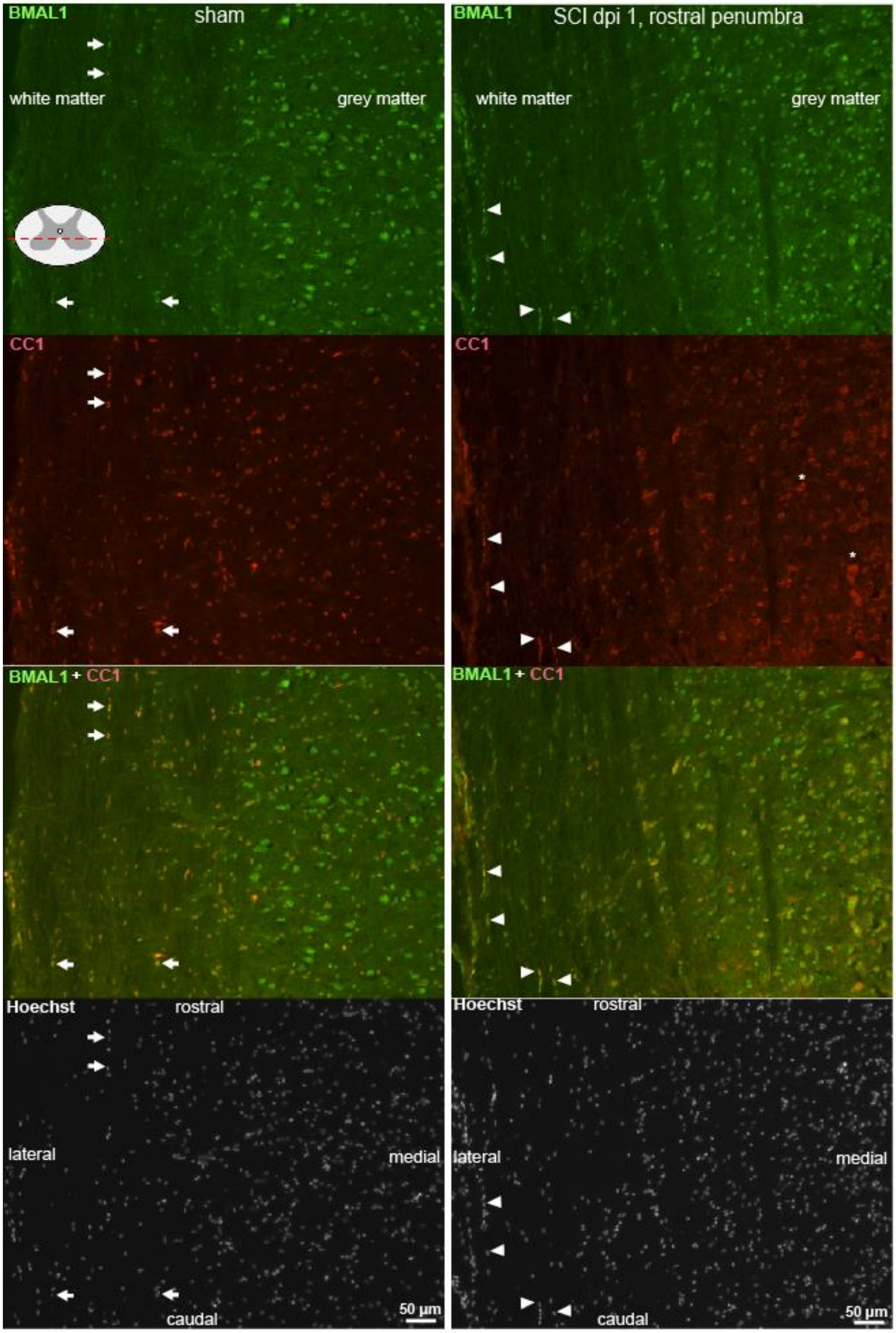

Slomnicki, Myers, Saraswat-Ohri et al., Fig. S4

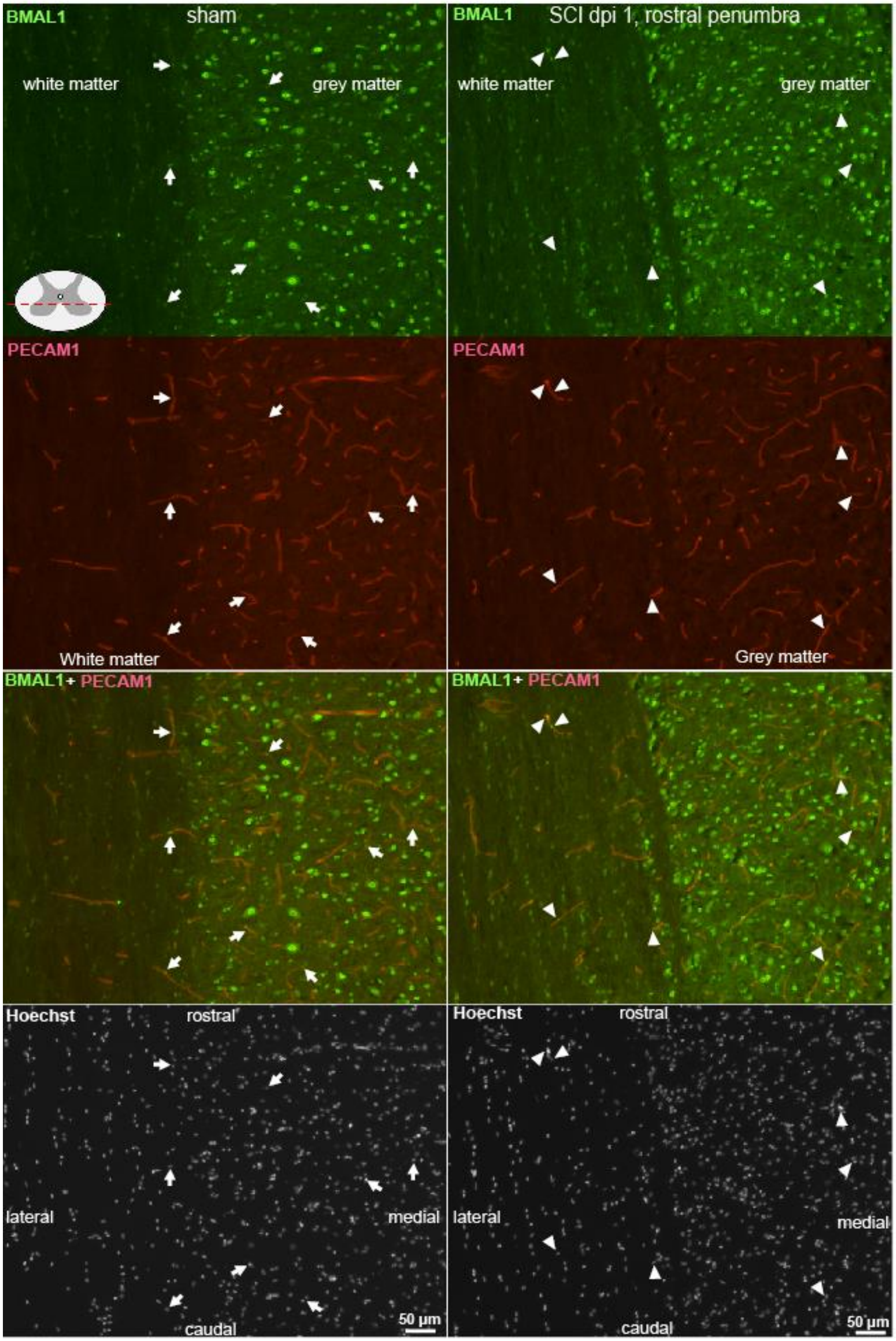

Slomnicki, Myers, Saraswat-Ohri et al., Fig. S5

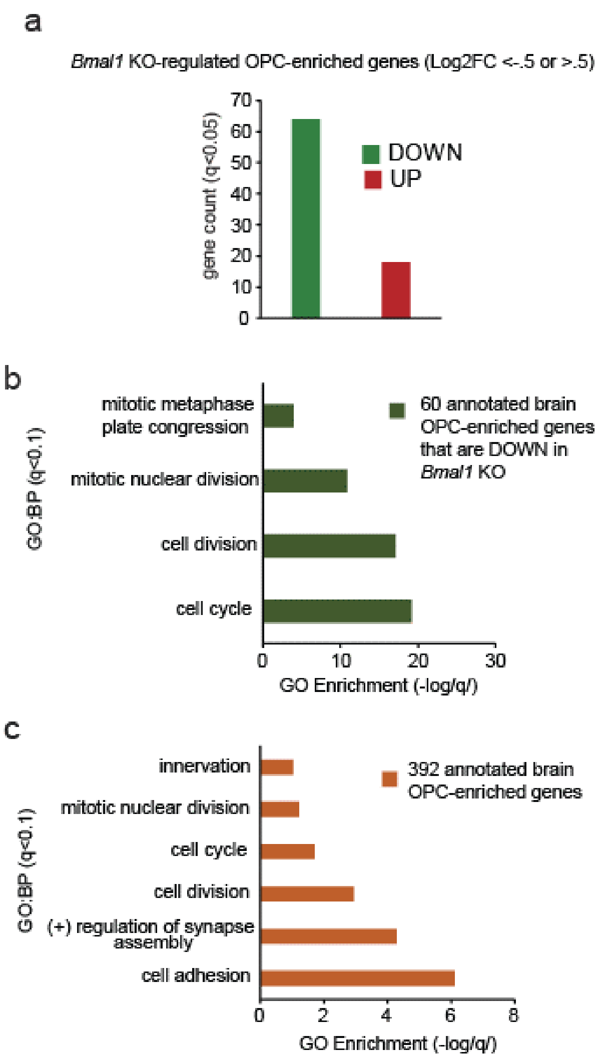

Slomnicki, Myers, Saraswat-Ohri et al., Fig. S6

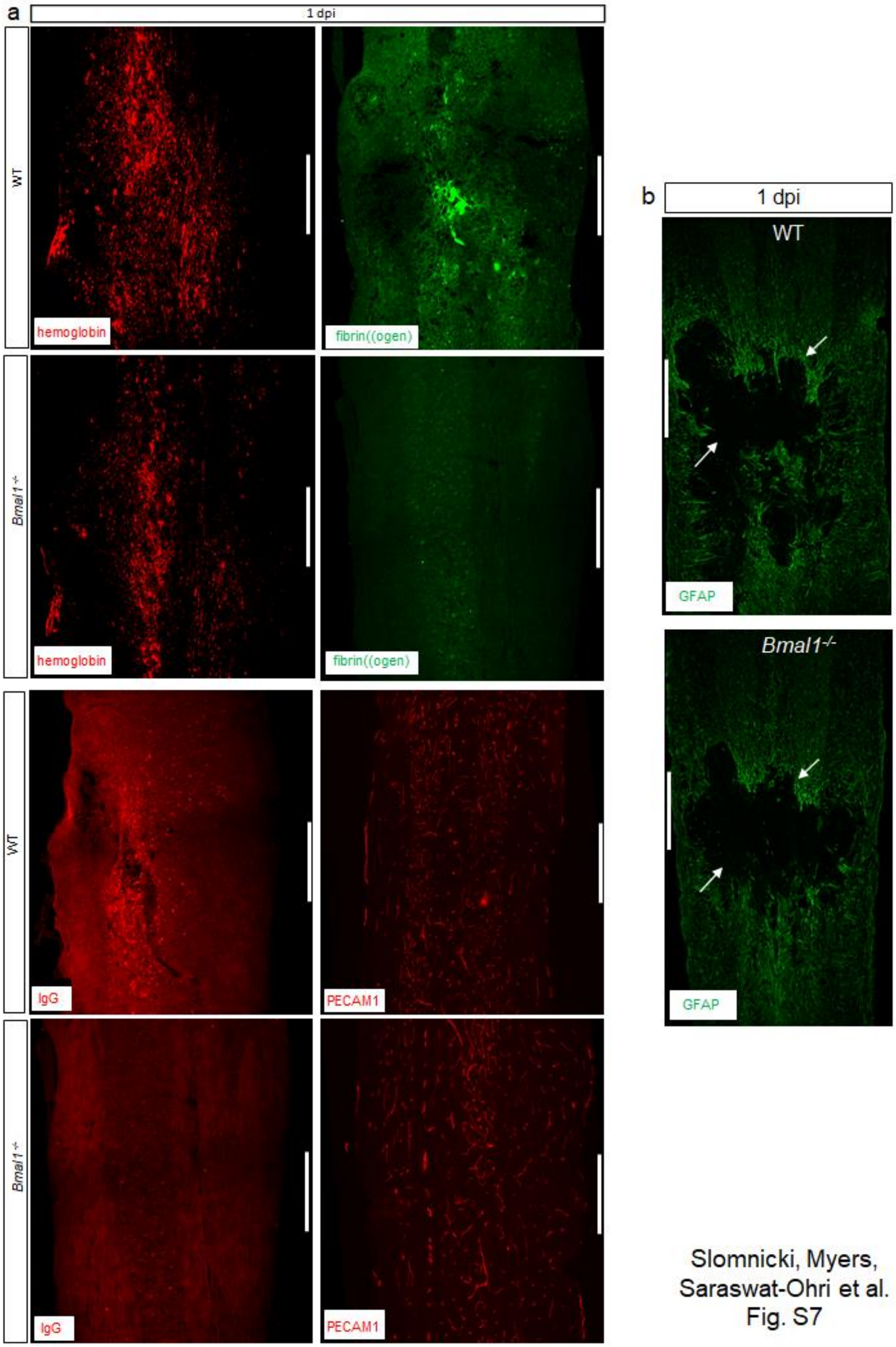

Slomnicki, Myers, Saraswat-Ohri et al. Fig. S7

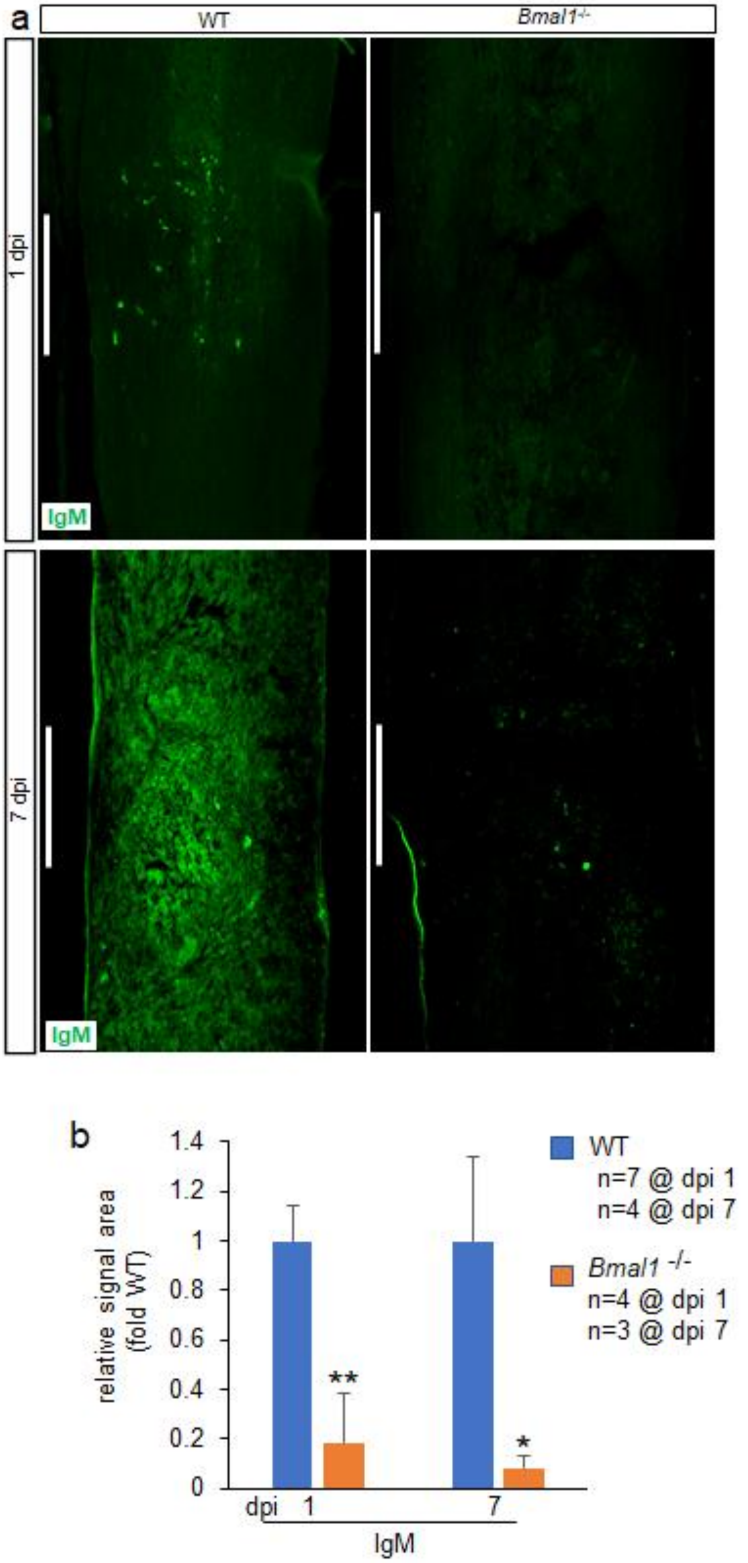

Slomnicki, Myers, Saraswat-Ohri et al. Fig. S8

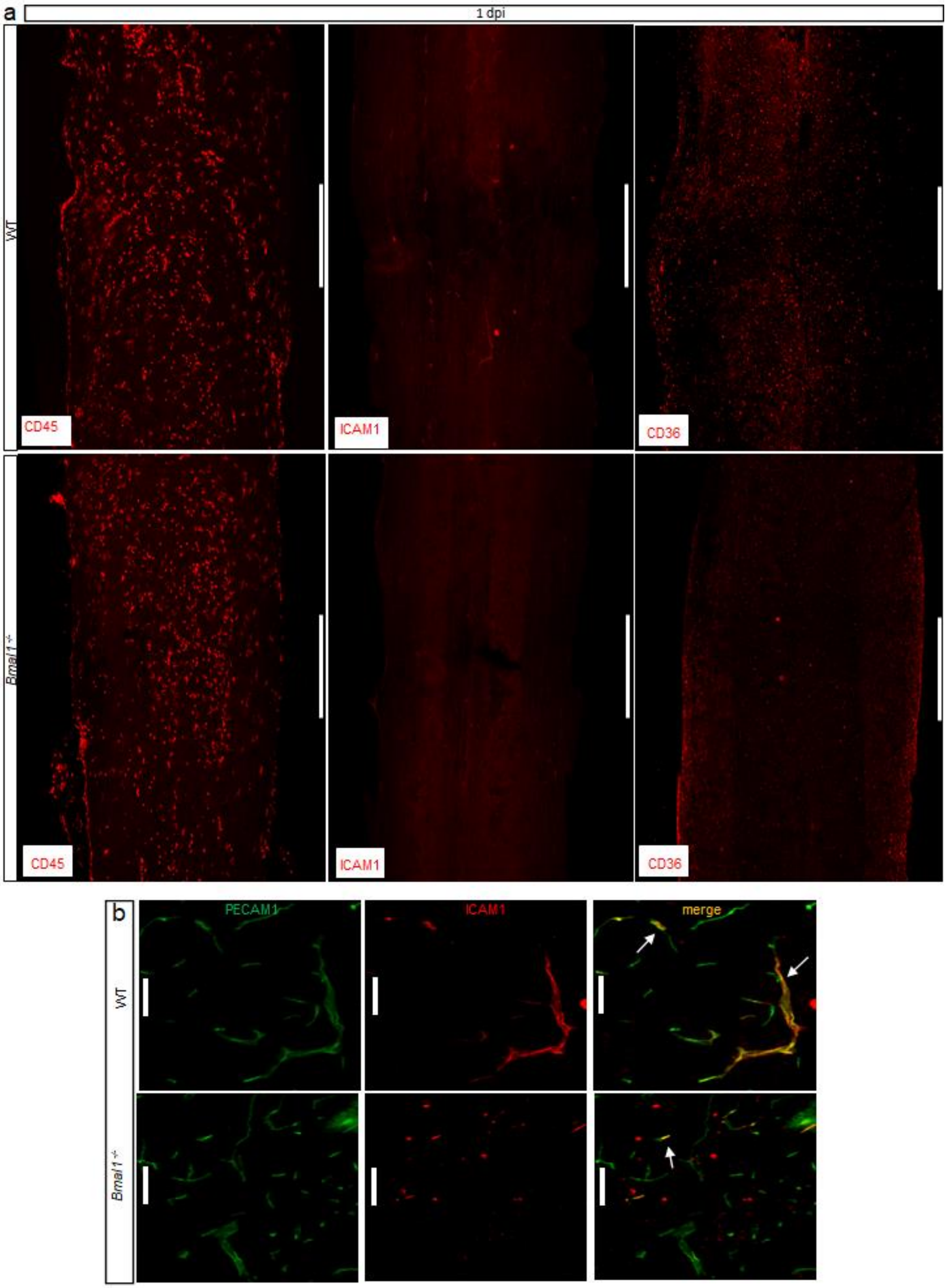

Slomnicki, Myers, Saraswat-Ohri et al. Fig. S9

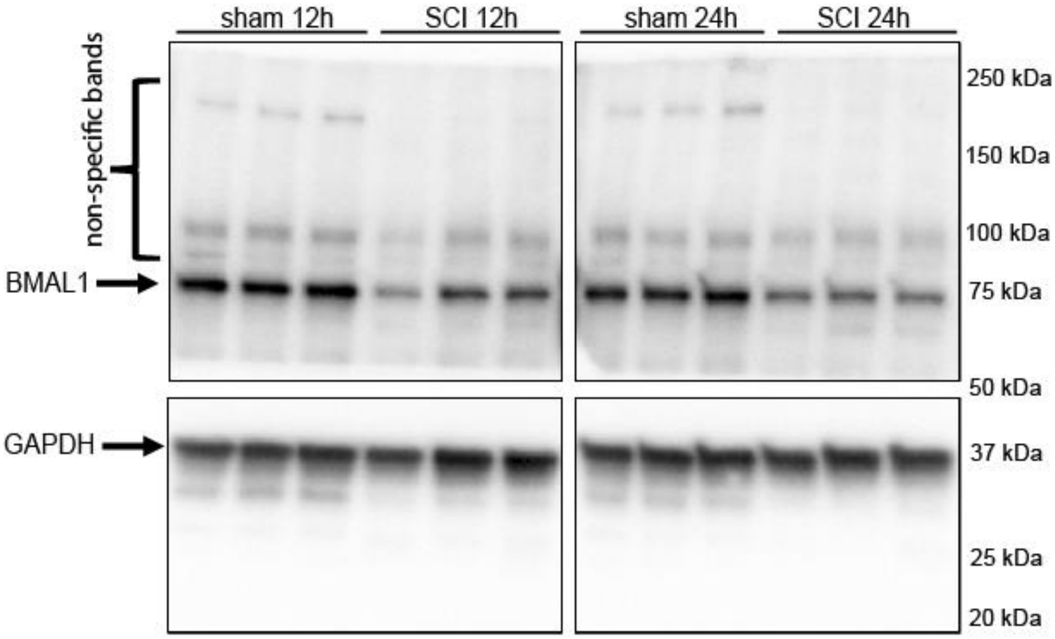

Slomnicki, Myers, Saraswat-Ohri et al., Fig. S10

Table S1. List of mRNAs whose expression is altered in *Bmal1*$^{-/-}$ mice on dpi 3 (a separate MS Excel spreadsheet is provided).

Table S2. Experimental design

Study (figure panel)	Groups	N (M:F)	Dual sample use for other studies
Analysis of clock pathway mRNAs after SCI (fig 1a)	WT sham@6h<br>WT sham@24h<br>WT SCI@6h<br>WT SCI@24h	2 (0:2)<br>3 (0:3)<br>4 (0:4)<br>4 (0:4)	None
Western blot analysis of BMAL1 expression after SCI (fig 1b, c)	WT sham@12h<br>WT sham@24h<br>WT SCI@12h<br>WT SCI@24h	3 (0:3)<br>6 (0:6)<br>3 (0:3)<br>6 (0:6)	None
Identification of BMAL1+ cells in sham or SCI spinal cord tissue using co-immunostaining (fig 2, 3 and S1-S5)	WT sham@24h<br>WT SCI@24h	3 (0:3)<br>3 (0:3)	None
Analyzing effects of *Bmal1*$^{-/-}$ genotype on clock pathway mRNAs after SCI (fig 4a)	WT SCI@dpi 3<br>*Bmal1*$^{-/-}$ SCI@dpi 3	5 (1:4) [a]<br>5 (1:4)[a]	Analyzing effects of *Bmal1*$^{-/-}$ genotype on acute loss of cell marker mRNAs (fig 5a)
Analyzing effects of *Bmal1*$^{-/-}$ genotype on locomotor recovery (fig 4b) [b]	WT SCI<br>*Bmal1*$^{-/-}$ SCI	14 (7:7)<br>10 (3:7)[a]	Analyzing WMS (fig 4c, d)<br>Analyzing chronic effects on OL content and axonal density (fig 5b-d)
Analyzing effects of *Bmal1*$^{-/-}$ genotype on WMS (fig 4c, d)	WT SCI@ dpi 42<br>*Bmal1*$^{-/-}$ SCI@ dpi 42	9 (5:4)<br>6 (2:4)[a]	None
Analyzing effects of *Bmal1*$^{-/-}$ genotype on OL content/axonal density (fig 5b-d)	WT SCI@ dpi 42<br>*Bmal1*$^{-/-}$ SCI@ dpi 42	5 (2:3) [a]<br>4 (1:3)[a]	None
Analyzing effects of *Bmal1*$^{-/-}$ genotype on the transcriptome of the injured spinal cord (fig 6, S6)	WT SCI@ dpi 3<br>*Bmal1*$^{-/-}$ SCI@ dpi 3	4 (2:2)<br>3 (1:2)[a]	None
Analyzing effects of *Bmal1*$^{-/-}$ genotype on markers of BSCB disruption and neuroinflammation (fig 7, 8, S7-S9)	WT SCI@ dpi 1<br>*Bmal1*$^{-/-}$ SCI@ dpi 1<br>WT SCI@ dpi 7<br>*Bmal1*$^{-/-}$ SCI@ dpi 7	7 (2:5) [a]<br>4 (0:4) [a]<br>4 (2:2)<br>3 (1:2)[a]	Analyzing effects of *Bmal1*$^{-/-}$ genotype on blood cell count and expression of selected coagulation factors on dpi 1 (Table S3, S3')

[a] in studies using *Bmal1*$^{-/-}$ mice unbalanced gender ratios were caused by (i) limited availability of males of comparable age, (ii) peri-operative loss of animals, and/or, (iii) initial genotyping errors that were later corrected by terminal genotyping resulting in group re-assignments.

[b] the study included two separate experiments using two batches of age-matched mice. For BMS analysis of locomotor recovery, data from both experiments were analyzed together. Tissues that were terminally collected from experiment 1 or experiment 2 (dpi 42) were used for WMS analysis or OL content/axonal density determination, respectively.

Supplementary Table S3. Complete blood count analysis at dpi 1: no significant differences between genotypes were detected.

Parameter	WT[a]	*Bmal1*$^{-/-}$ [b]
PLT*k/µl (platelets)	878.67 ± 24.5	832 ± 50.24
MPV*fL	6.8 ± 0.27	6.18 ± 0.18
RBC*M/µl	10 ± 1.67	9.5 ± 0.73
HGB [g/dL]	14.77 ± 2.1	14.43 ± 1.145
HCT [%]	49.57 ± 9.35	47.65 ± 4.45
WBC*k/µl	1.683 ± 0.279	2.533 ± 0.609
Neut*k/µl	0.373 ± 0.165	0.725 ± 0.283
Lymph*k/µl	1.207 ± 0.148	1.73 ± 0.37
Mono*k/µl	0.08 ± 0.02	0.065 ± 0.01
Eo*k/µl	0.02 ± 0.01	0.013 ± 0.005
Baso*k/µl	0	0
MCV [fL]	49.2 ± 1.32	50 ±0.76
MCH [pg]	14.9 ± 0.42	15.18 ± 0.1
MCHC [g/dl]	30.4 ± 1.65	30.38 ± 0.39
RDW-SD [fL]	29.4 ± 1.74	30.15 ± 1.41
RDW-CV [%]	23.93 ± 2.13	23.43 ± 1.08
RET [K/µL]	428.3 ± 56.94	354.425 ± 13.66
PDW [fL]	8.67 ± 0.47	7.5 ± 0.36
P-LCR [%]	6.73 ± 1.14	3.825 ± 0.55
PCT [%]	0.603 ± 0.037	0.515 ± 0.047

Supplementary Table S3'. qPCR analysis of liver RNA at dpi 1 (all expression levels normalized to *Gapdh*): in KOs, *Bmal1* mRNA is undetectable and *Nr1d1* is low.

Gene	WT (fold change WT)	*Bmal1*$^{-/-}$ (fold change WT)
F7	1 ± 0.174	0.78 ± 0.13
Vwf	1 ± 0.21	0.83 ± 0.12
Fga (fibrinogen α)	1 ± 0.244	1.252 ± 0.218
Fgb (fibrinogen β)	1 ± 0.228	1.533 ± 0.199
Fgg (fibrinogen γ)	1 ± 0.235	2.156 ± 0.625
Bmal1/Arntl	1 ± 0.189	undetectable
Nr1d1	1 ± 0.135	**0.044 ± 0.009***

[a] n=3, all F, all littermates of *Bmal1*$^{-/-}$ mice

[b] n=4, all F

***** p<0.05, *u*-test

Supplementary Table S4. List of primary antibodies for immunofluorescence.

Antibody	Origin	Dilution	Company	Catalog#
anti-BMAL1	Rabbit	1:500	Novus Biologicals	NB100-2288
anti-APC (CC-1)	Mouse	1:100	Calbiochem	OP80
anti-CD31 (PECAM1)	Rat	1:200	BD Pharmaceutical	550274
anti-GFAP	Chicken	1:200	Millipore	AB5541
anti-NeuN	Mouse	1:200	Millipore	MAB377
anti-Neurofilament H (NFH)	Chicken	1:500	Millipore	AB5539
anti-hemoglobin α (HBA1+2)	rabbit	1:150	LifeSpan BioSciences	LS-C409143
anti-fibrinogen (α chain)	rabbit	1:150	Bioss Antibodies	bs-7548R
anti-CD45 (IBL-5/25)	rat	1:150	Millipore	CBL1326
anti-CD36 (IgA; CRF-D2712)	mouse	1:100	BD Pharmingen	552544
anti-CD54 (ICAM, clone 3E2)	hamster	1:100	BD Pharmingen	550287
anti-Von Willebrand factor	sheep	1:100	Novus Biologicals	NB100-62174
anti-IgG	donkey	1:200	Jackson ImmunoResearch	715-546-150
anti-IgM	donkey	1:100	Jackson ImmunoResearch	715-156-020
Rabbit IgG control		1:500	Novus Biologicals	AB-105-C
Mouse IgG control		1:100	Invitrogen	08-6599
Hamster IgG control		1:100	Invitrogen	HM00
Rat IgG control		1:150	Abcam	ab37361
Sheep IgG control		1:100	Abcam	ab37385

Supplementary Table S5. List of qPCR primers.

Gene	Assay ID (ThermoFisher Scientific TaqMan Assay)
Map2	Mm00485230_m1
Eno2	Mm00469062_m1
Mbp	Mm01266402_m1
Plp1	Mm01297210_m1
Nos3	Mm00435217_m1
Ccl2	Mm00441242_m1
Ptgs2	Mm00478374_m1
Adamts8	Mm00479220_m1
Aplnr	Mm00442191_s1
Ptgis	Mm00447271_m1
Slco2a1	Mm00459638_m1
F7	Mm00487329_m1
Vwf	Mm00550376_m1
Fga	Mm00802584_m1
Fgb	Mm00805336_m1
Fgg	Mm00513575_m1
**Gene**	**Primer sequences used for SybrGreen reactions**
Arntl	For: 5'-GCAGTGCCACTGACTACCAAGA-3'<br>Rev: 5'-TCCTGGACATTGCATTGCAT-3'
Cry1	For: 5'-CCCAGGCTTTTCAAGGAATGGAACA-3'<br>Rev: 5'-TCTCATCATGGTCATCAGACAGAGG-3'
Per1	For: 5'-TCCTCCTCCTACACTGCCTCT-3'<br>Rev: 5'-TTGCTGACGACGGATCTTT-3'
Nr1d1	For: 5'-ACGACCCTGGACTCCAATAA-3'<br>Rev: 5'-CCATTGGAGCTGTCACTGTAGA-3'
Dbp	For: 5'-AATGACCTTTGAACCTGATCCCGCT-3'<br>Rev: 5'-GCTCCAGTACTTCTCATCCTTCTGT-3'
